# Longitudinal Changes in Cardiovascular-Kidney-Metabolic Syndrome Stages and Their Impact on Outcomes: A Nationwide Cohort Study

**DOI:** 10.3390/jcm14113888

**Published:** 2025-06-01

**Authors:** Byung Sik Kim, Hyun-Jin Kim, Hasung Kim, Jungkuk Lee, Jeong-Hun Shin, Ki-Chul Sung

**Affiliations:** 1 Division of Cardiology, Department of Internal Medicine, Hanyang University Guri Hospital, Hanyang University College of Medicine, Guri 11923, Republic of Korea; fish3777@hanmail.net (B.S.K.); titi8th@gmail.com (H.-J.K.); 2 Data Science Team, Hanmi Pharm. Co., Ltd., Seoul 05545, Republic of Korea; hasung@hanmi.co.kr (H.K.); jungkuk.lee@hanmi.co.kr (J.L.); 3 Division of Cardiology, Department of Internal Medicine, Kangbuk Samsung Hospital, Sungkyunkwan University School of Medicine, Seoul 06351, Republic of Korea

**Keywords:** cardiovascular diseases, kidney diseases, metabolic diseases, outcome studies

## Abstract

**Background/Objectives:** The impact of longitudinal changes in cardiovascular-kidney-metabolic (CKM) stage remains unclear. This study evaluated the association between CKM stage progression and clinical outcomes. **Methods**: We used the Korean National Health Insurance Database to identify adults aged ≥ 20 years who underwent two health checkups between 2009 and 2012. CKM stages were assessed at both time points and categorized as decreased, maintained, or increased over a 1–2-year interval. The primary outcome was a composite of all-cause death, heart failure, stroke, and myocardial infarction, evaluated over a mean follow-up of 11.05 years. **Results**: Among 877,537 participants, 15.3% experienced CKM stage progression. Compared to the maintained group, the increased group had a higher risk of the composite outcome (HR: 1.071, 95% CI: 1.050–1.092). While men had a higher rate of progression, women showed greater risk of clinical events (HR: 1.124 vs. 1.040). While stage progression was more frequent in younger adults, older individuals in the increased group progressed to more advanced stages and experienced higher rates of adverse outcomes. **Conclusions**: CKM stage progression is independently associated with increased risk of mortality and cardiovascular events, particularly in women and older adults. Serial CKM assessment may help identify high-risk individuals for early intervention.

## 1. Introduction

Cardiovascular-kidney-metabolic (CKM) syndrome involves an interplay between cardiometabolic disorders, kidney dysfunction, and cardiovascular diseases [1]. It is a progressive condition, beginning from a healthy state (stage 0), in which early-life exposures trigger adipose tissue dysfunction, inflammation, oxidative stress, and insulin resistance, eventually leading to chronic kidney disease (CKD) and cardiometabolic risk factors such as hypertension, hypertriglyceridemia, abdominal obesity, and diabetes mellitus. Over time, these interconnected conditions progress to subclinical coronary atherosclerosis, subclinical myocardial dysfunction, and declining kidney function, ultimately causing clinical cardiovascular disease (CVD), kidney failure, disability, and premature death; consequently, these changes warrant early detection and intervention [2,3]. This framework provides a foundation for enhancing risk stratification and implementing targeted interventions to mitigate the cumulative burden of these interrelated conditions [1,2].

Static assessments of CKM stage provide valuable clinical information but do not capture dynamic changes in disease stage that provide insights into disease progression that can guide prevention. Cardiovascular risk prediction algorithms, such as the Systematic Coronary Risk Evaluation of the European Society of Cardiology and the Atherosclerotic Cardiovascular Disease (ASCVD) risk calculator and seven cardiovascular health metrics (Life’s Simple 7), both proposed by the American Heart Association (AHA), primarily rely on static risk factor measurements. However, incorporating longitudinal changes in these scores enhances predictive accuracy [4,5,6].

Scarce evidence exists regarding the association between temporal changes in CKM syndrome stage and cardiovascular outcomes. Here, we aimed to evaluate such relationships to improve risk stratification and inform targeted intervention strategies.

## 2. Materials and Methods

### 2.1. Study Design and Patient Population

This retrospective cohort study utilized data from the Korean National Health Insurance Database (NHID), a comprehensive national resource encompassing healthcare information for nearly the entire Korean population. Detailed information about the NHID has been reported previously [7]. The NHID includes detailed medical records, such as diagnoses, treatments, prescriptions, and mortality, as well as data from routine health screening programs conducted biennially for eligible individuals. The health screening programs, initiated to support early detection and prevention of chronic diseases collect data on various health indicators, including lifestyle factors, clinical measurements, and laboratory results. These programs are integrated into the NHID, providing a robust dataset for longitudinal health research.

**Figure 1:**
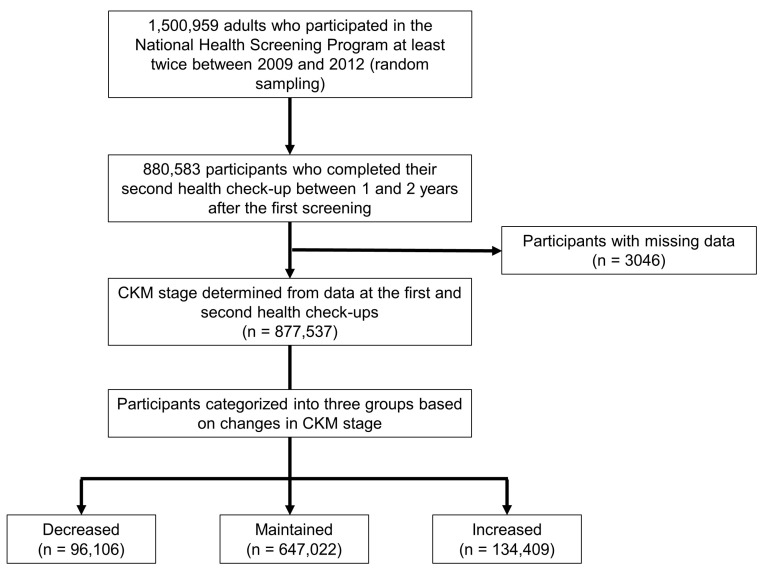
Study flowchart.

This study included adults aged ≥ 20 years who underwent two health check-ups within a period of 1–2 years (2009–2012). From an initial sample of 1,500,959 individuals selected through stratified random sampling, 880,583 participants met the inclusion criteria (completion of their second health check-up within 1–2 years after the first screening). Patients with missing data were excluded (n = 3046), resulting in a final cohort of 877,537 individuals. The CKM stage was assessed during both health check-ups, and participants were categorized into three groups based on CKM stage changes over time, either the decreased (n = 96,106), maintained (n = 647,022), or increased (n = 134,409) stage groups. These groups were analyzed to evaluate their association with subsequent clinical outcomes (Figure 1).

This study was approved by the Institutional Review Board of our facility (Ethical Approval Number: GURI 2024-12-021). Informed consent was waived because the NHID dataset is publicly available, anonymized, and devoid of personally identifiable information.

### 2.2. Data Collection

The collected data included demographic characteristics (age and sex), lifestyle behaviors (smoking status, alcohol intake, and physical activity), and comprehensive anthropometric and laboratory measurements. Smoking status was classified into the following three categories: never, past, and current. Alcohol consumption was grouped into none, 1–2 times per week, 3–4 times per week, or ≥5 times per week categories. Physical activity levels were categorized by frequency into 0, 1–2, 3–4, 5–6, or 7 sessions per week. Anthropometric measurements included body mass index (BMI), which was categorized as underweight (<18.5 kg/m^2^), normal weight (18.5–22.9 kg/m^2^), overweight (23.0–24.9 kg/m^2^), or obese (≥25 kg/m^2^), following Asian-specific criteria established by the Korean Society for the Study of Obesity and the World Health Organization Asia-Pacific definition [8]. Waist circumference was also measured to assess central obesity. Blood pressure (BP) was measured using sphygmomanometers or oscillometric devices after 3 to 5 min of rest at health examination centers or clinics. Measurements were taken at least twice at 1–2 min intervals by trained medical personnel using appropriately sized cuffs [9]. Laboratory data included fasting glucose, total cholesterol, high-density lipoprotein cholesterol, low-density lipoprotein cholesterol, triglycerides, and estimated glomerular filtration rate (eGFR). Socioeconomic status was determined using household income quartiles based on monthly health insurance premiums. Clinical information included a history of hypertension, diabetes mellitus, and dyslipidemia, identified through medical records and prescriptions. The use of specific medications was also documented, including antihypertensive drugs, glucose-lowering drugs, lipid-lowering medications, and antiplatelet drugs. Additionally, the AHA-derived predicting risk of cardiovascular disease events (PREVENT) score was calculated to provide an integrated assessment of cardiovascular and metabolic risk [10].

### 2.3. Definition and Staging of CKM Syndrome

CKM syndrome stages were classified using clinical and laboratory parameters that reflect metabolic, cardiovascular, and renal health, as outlined in the AHA Scientific Statement [1]. In this study, to reflect the characteristics of the Korean population, we applied Asian-specific cut-off values for BMI and components of metabolic syndrome (waist circumference and BP) based on Korean guidelines [8,11]. Stage 0 represented individuals with optimal health, characterized by not being overweight or obese, metabolic risk factors such as hypertension, hypertriglyceridemia, and metabolic syndrome, or diabetes mellitus, CKD, or subclinical or clinical CVD. Stage 1 encompassed individuals with excess or dysfunctional adiposity, including being overweight or exhibiting abdominal obesity, without evidence of additional metabolic or renal dysfunction. Participants in stage 2 exhibited metabolic risk factors, such as hypertension, hypertriglyceridemia, metabolic syndrome, diabetes, or CKD defined by an eGFR of 30–59 mL/min/1.73 m^2^. Stage 3 included risk equivalents of subclinical CVD, such as very-high-risk CKD, defined as an eGFR < 30 mL/min/1.73 m^2^, or a high predicted 10-year CVD risk, exemplified by a PREVENT score ≥ 20% [12]. Stage 4 represented individuals with clinical manifestations of CVD, including coronary heart disease, heart failure, stroke, peripheral artery disease, or atrial fibrillation, often occurring in the context of excess or dysfunctional adiposity, metabolic risk factors, or advanced CKD. A comprehensive description of the CKM staging criteria is provided in Table 1.

### 2.4. Study Outcomes

The second health check-up was used as the index date, and clinical outcomes were assessed from this point forward. The primary outcome was defined as the first occurrence of a composite of all-cause death, heart failure, stroke (ischemic or hemorrhagic), and myocardial infarction (MI) during the follow-up period ending 31 December 2022. The mean follow-up duration was 11.05 ± 1.49 years, with a median of 13.02 years (interquartile range: Q1 12.25 years and Q3 13.39 years). Secondary outcomes included each component of the primary outcome analyzed individually. Heart failure was defined as hospitalization with *International Classification of Diseases, Tenth Revision* (ICD-10) codes I50, I42.0, I11.0, or I13.0-I13.2. MI was identified as hospitalized patients who underwent coronary revascularization and had discharge diagnoses coded as I21 or I22. Stroke was defined as patients with hospital admissions where brain imaging was performed, and discharge diagnoses included ICD-10 codes I63–I64 for ischemic stroke or I60–I62 for hemorrhagic stroke. A comprehensive list of diagnostic and procedural definitions, including the corresponding ICD-10 codes, used for defining comorbidities, CKM staging, and clinical outcomes, is presented in Table S1 [13].

### 2.5. Statistical Analysis

Baseline characteristics across CKM stage-change groups (decreased, maintained, and increased) were compared using Chi-square tests for categorical variables and analysis of variance for continuous variables. The distribution of CKM stage changes between the first and second health check-ups was visualized using Sankey diagrams, illustrating the dynamic transitions between CKM stages over time. Outcome incidence rates were expressed as events per 1000 person-years, and Cox proportional hazards regression models were applied to estimate hazard ratios (HRs) and 95% confidence intervals (CIs) for both primary and secondary outcomes, with the “maintained” stage group serving as the reference category. To account for potential confounders, the models were adjusted in three steps. The first model included adjustments for age and sex, while the second model further incorporated lifestyle factors such as smoking status, alcohol consumption, and physical activity, along with socioeconomic status represented by household income. The final model adjusted for medication use, including antihypertensive, glucose-lowering, lipid-lowering, and antiplatelet drugs. Kaplan–Meier curves were constructed to visualize survival probabilities across the CKM stage-change groups, and statistical differences were assessed using the log-rank test. Adjusted survival curves were generated using direct adjustment methods based on the covariates included in the final model to provide a clearer understanding of the cumulative risk associated with each CKM stage-change group [14]. Subgroup analyses were performed to evaluate whether the associations between CKM stage changes and clinical outcomes varied by age and sex. Using the same modeling approach as the primary analysis, Cox proportional hazards regression models were stratified by sex (men and women) and age categories (20s, 30s, 40s, 50s, and ≥60s) to estimate HRs and 95% CIs for the primary and secondary outcomes within these subgroups. Interaction terms between CKM stage changes and subgroup variables (age and sex) were included to test for potential effect modification. Statistical significance was defined as a two-sided *p*-value < 0.05. All analyses were conducted using SAS software (version 9.4; SAS Institute) and R software (version 4.2.1; R Foundation for Statistical Computing).

## 3. Results

### 3.1. Baseline Characteristics

Baseline characteristics stratified by CKM stage-change groups (decreased, maintained, and increased) are summarized in Table 2. The majority of participants belonged to the maintained CKM stage group (73.7%), followed by the increased (15.3%) and decreased (11.0%) stage groups. Significant differences were observed across groups for demographic, clinical, and lifestyle factors. Participants in the maintained group were older and included a higher proportion of women (49.43 ± 13.91 years, 45.40%) compared to the decreased (45.20 ± 13.12 years, 41.42%) and increased (46.04 ± 14.00 years, 39.70%) groups (*p* < 0.001). The maintained stage group had the highest prevalence of hypertension, diabetes, and dyslipidemia. Furthermore, medication use, including antihypertensive drugs, glucose-lowering drugs, and lipid-lowering drugs, was highest in this group. Participants in the increased group exhibited higher BP, fasting glucose, and triglycerides levels compared to the other groups. This group also had the highest PREVENT scores (6.92 ± 10.57 vs. 6.05 ± 8.07 vs. 3.33 ± 4.87, *p* < 0.001). The proportion of current smokers was highest in the increased stage group.

### 3.2. Changes in CKM Stages Between Health Check-Ups

**Figure 2:**
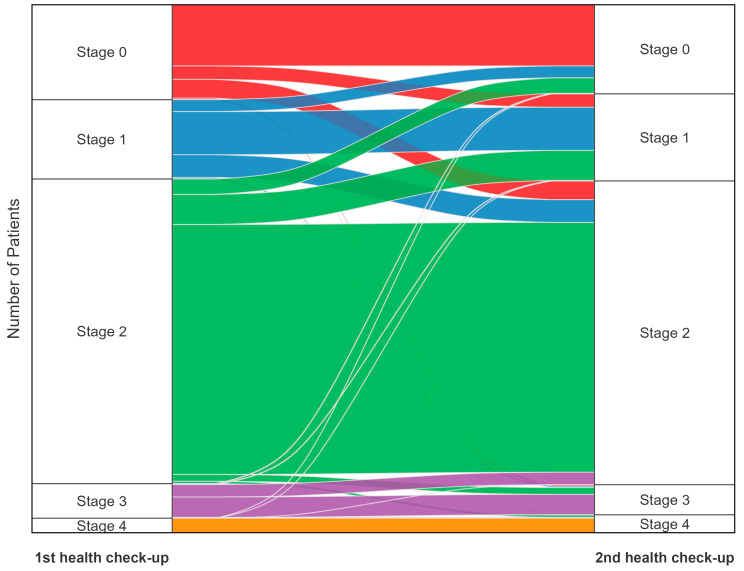
CKM stage changes between health check-ups.

The distribution of CKM stage changes between the first and second health check-ups is depicted in Figure 2 and Tables S2–S4. Most stage 0 participants (64.6%) remained in stage 0, while a substantial proportion progressed to stage 1 (14.1%) or stage 2 (19.7%). Among those initially categorized as stage 1, 34% advanced to stage 2, representing the highest observed upward transition rate. Meanwhile, in the stage 2 group, which includes the largest number of participants, 12.6% experienced a transition to a lower stage (stage 1 or stage 0). Overall, 3.53% of all participants progressed to stage 3 or 4, accounting for 23.08% of those who exhibited a stage increase. Subgroup analysis by sex (Figure S1A,B) revealed distinct differences in CKM stage transitions. Men were more likely than women to transition to higher stages (16.5% vs. 13.8%). Men also exhibited a higher progression rate to stage 3 or 4 (4.22%) compared to women (2.67%). Age group analysis (Figure S1C–G) revealed distinct trends in CKM stage transitions. Younger participants (20–29 years) exhibited higher upward transitions, with 72.2% of stage 0 individuals remaining at stage 0 while 13.8% progressed to stage 1 and 13.0% to stage 2. In contrast, progression to stage 3 or 4 was uncommon in younger individuals (2.22% in aged 20–29 years) but more frequent in older adults, particularly those aged ≥ 60 years (9.04%).

### 3.3. Clinical Outcomes According to Changes in CKM Stage

**Figure 3:**
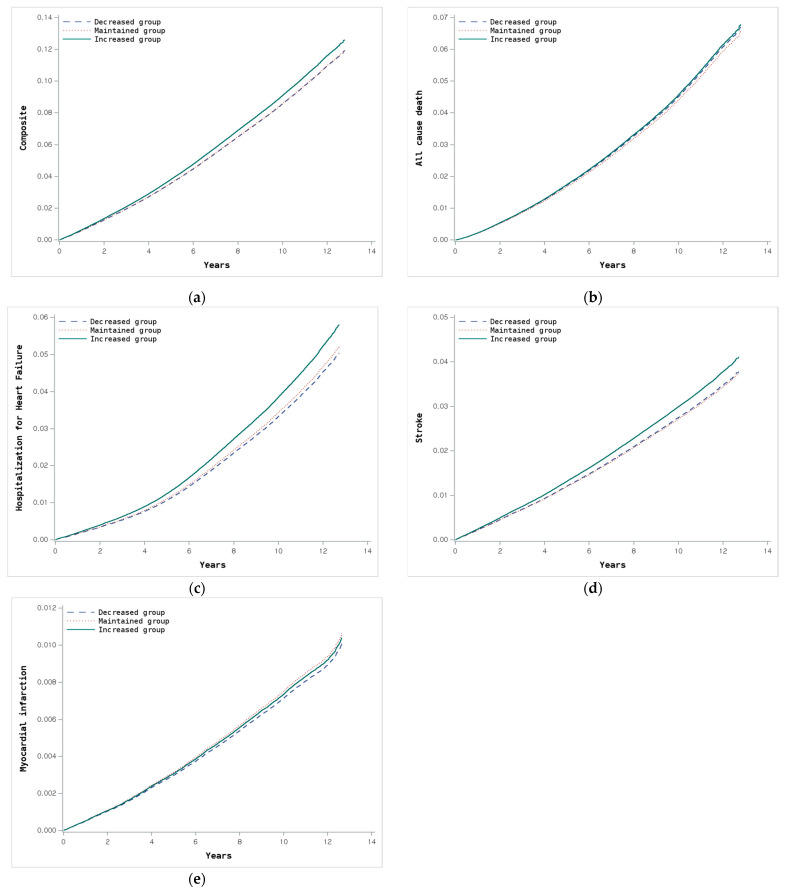
Adjusted event curves according to changes in CKM stage. (**a**) Composite primary outcome (all-cause death, heart failure, stroke, or myocardial infarction); (**b**) all-cause death; (**c**) heart failure; (**d**) stroke (ischemic or hemorrhagic); and (**e**) myocardial infarction.

The clinical outcomes associated with changes in CKM stage are summarized in Table 3 and visualized through Kaplan–Meier curves (Figure S2) and adjusted survival curves (Figure 3). The incidence rate of the composite primary outcome, including all-cause death, heart failure, stroke, or MI, was highest in the maintained stage group (10.03 per 1000 person-years), followed by the increased stage (8.42 per 1000 person-years) and the decreased stage (6.27 per 1000 person-years) groups. After adjusting for potential confounding factors, participants in the increased group demonstrated a significantly higher risk for the composite primary outcome (HR 1.071, 95% CI 1.050–1.092, and *p* < 0.001) compared to the maintained group. In contrast, the decreased stage group showed no significant difference in risk after full adjustment (HR 0.997, 95% CI 0.972–1.024, and *p* = 0.849). For individual outcomes, the increased stage group demonstrated significantly higher risks across all endpoints compared to the maintained group, except for myocardial infarction which showed no significant difference. In contrast, the decreased stage group did not exhibit significant differences in risk for any of the individual outcomes after full adjustment.

### 3.4. Age and Sex Differences in Clinical Outcomes According to Changes in CKM Stage

The subgroup analysis of composite primary outcomes by sex and age is summarized in Table 4, with significant interaction effects observed for both sex (interaction *p* < 0.001) and age groups (interaction *p* < 0.001). After adjusting for confounding factors, the increased stage group showed a higher risk compared to the maintained stage group in both men and women. However, the HR was notably higher in women (HR 1.124, 95% CI 1.088–1.161, and *p* < 0.001) than in men (HR 1.040, 95% CI 1.016–1.066, and *p* = 0.001). In contrast, no significant differences were observed in the decreased stage group for either sex. Age-specific trends were evident, with older age groups exhibiting higher event rates overall. For participants aged 20–29, 30–39, and 40–49 years, no significant differences were observed between CKM stage-change groups after adjustment. In contrast, older age groups (50–59 and ≥60 years) showed significantly higher risks in the increased stage group, with the 50–59 age category exhibiting a particularly elevated risk (HR 1.096, 95% CI 1.070–1.123, and *p* < 0.001). For individual outcomes, such as all-cause death, heart failure, stroke, and MI, similar patterns were observed. The increased stage group consistently exhibited higher risks compared to the maintained stage group, while no significant differences were noted in the decreased stage group across most outcomes (Tables S5–S11).

### 3.5. Clinical Outcomes According to Baseline CKM Stage

We further evaluated clinical outcomes according to changes in CKM stage stratified by baseline stage (Tables S12–S15). Across all baseline stages (0 to 3), participants who experienced stage progression exhibited a significantly higher risk of the composite outcome compared to those who maintained their stage. In contrast, among individuals at baseline stage 2 or 3, regression to a lower stage was associated with a reduced risk of adverse events. These trends were consistent across individual outcomes, including all-cause death, heart failure, stroke, and myocardial infarction.

## 4. Discussion

This was the first large-scale study to investigate the association between longitudinal changes in CKM stage and clinical outcomes, utilizing data from a nationwide database. Our findings reveal the importance of longitudinal CKM stage assessments in identifying participants at elevated risk for adverse outcomes (all-cause death, heart failure, stroke, and MI). The key findings are as follows: (1) We observed significant shifts in CKM staging among adults over 1–2 years: 11% exhibited a lowered while 15.3% displayed an elevated stage. Notably, among those initially at stage 1, considered a relatively lower-risk group, 34% advanced to stage 2. In addition, 12.6% of the largest group (stage 2) transitioned to stage 1 or 0. (2) Participants in the increased CKM stage group demonstrated a significantly higher risk of composite primary outcomes compared to those in the maintained group, while no significant differences were observed in the decreased group. (3) Age and sex modified the association between CKM stage changes and clinical outcomes, with women and adults aged ≥ 50 years exhibiting particularly elevated risks in the increased group. (4) In baseline stage-stratified analyses, CKM stage progression was consistently associated with increased clinical risk across all stages, while stage regression in stages 2 and 3 was linked to reduced risk.

CKM syndrome is significantly associated with adverse outcomes [3,15], and our current findings further expand this association. Among the increased CKM stage group, 23.08% (3.53% of the total cohort) progressed to stage ≥ 3. Additionally, among those who were initially at stage 1, 34% progressed to stage 2, representing the highest observed transition rate and warranting vigilant monitoring despite its lower baseline risk. These finding highlights that CKM syndrome is not a static condition but rather a dynamic process characterized by transitions between stages over time. Moreover, our findings align with prior studies showing that longitudinal assessments of cardiovascular health indices are robust predictors of future cardiovascular adverse events [4,5].

We observed significant age- and sex-specific differences in CKM stage transitions and associated risks. Women in the increased stage group had an elevated adverse outcome risk compared with that of men (HR: 1.124 vs. 1.040, respectively), although men exhibited a higher incidence of stage progression than women (16.52% vs. 13.79%, respectively). This finding aligns with prior reports indicating that although women compared with men tend to have a lower prevalence of advanced CKM stages, they experience higher mortality risk across the CKM spectrum [16]. While prior studies focused on prevalence trends and primarily addressed all-cause mortality, our longitudinal approach provides additional insight into the dynamic nature of CKM stage transitions and extends the analysis to include cardiovascular events.

We also studied age-related CKM stage progression and found that younger patients had a higher prevalence of stage increase (20.36% in those aged in the 20 s vs. 12.63% in those aged ≥ 60). However, older individuals (aged 50–59 and ≥60 years) in the increased stage group were more likely to progress to advanced stages (71.52% for age ≥ 60) and experience adverse outcomes, emphasizing the importance of age- and sex-based intervention [17,18]. For younger populations lifestyle interventions targeting early-life risk factors may prevent CKM progression, whereas older adults require aggressive management of advanced-stage risk factors to mitigate compounded risks.

Our present data have significant implications for clinical practice. First, CKM staging identifies patients at high risk and stratifies risk via longitudinal CKM stage changes [19]. It moves beyond static risk prediction and employs temporal stage changes to manage disease progression and intervention. For example, even individuals in the early stages may benefit from targeted evaluation if their CKM stage progresses over time. In such cases, comprehensive evaluations—such as those recommended by current clinical guidelines for cardiovascular risk stratification—may be considered to detect asymptomatic hypertension-mediated organ damage and subclinical ASCVD [1,20]. Innovative imaging modalities, such as speckle tracking echocardiography, also play an important role in early CKM stages, particularly for detecting subtle impairments in myocardial strain before overt symptoms appear [21,22]. These tools can help identify candidates for early initiation or intensification of cardioprotective therapies before clinical events occur. Second, members of the increased stage group are a high-priority cohort for intensive risk factor management and therapies (BP control, glycemic management, and lipid-lowering therapy). Third, sex- and age-based disparities warrant interventions tailored to these subgroups. Women transitioning to higher CKM stages need metabolic risk factor management whereas older adults require comprehensive approaches addressing multimorbidity.

This study has several strengths, including the use of longitudinal data from a large, nationwide cohort with rigorous statistical adjustments. Integrating dynamic CKM staging offers novel insights into the progression of metabolic, cardiovascular, and renal risks. However, this study has several limitations. First, it relied on health insurance claims data, which may lead to misclassification or inaccuracies in diagnoses, although these are mitigated by the large sample size and validated definitions. Second, CKM staging was determined using parameters from routine health check-ups, which may be sensitive to minor fluctuations. For example, small variations in eGFR near threshold values could result in changes in CKM stage classification. However, this situation may reflect real-world clinical practice. Third, the staging system may not fully capture subclinical ASCVD or myocardial dysfunction due to limitations in available screening data. Fourth, we were unable to account for changes in medication use, such as antihypertensive, antidiabetic, or lipid-lowering therapies, during the follow-up period, which may have influenced clinical outcomes. Fifth, although CKM stage was assessed twice between 2009 and 2012, subsequent changes in CKM status after this period were not captured. As progression or improvement could have occurred during the long follow-up, this limits our ability to interpret the dynamic nature of CKM staging over time. Sixth, although the use of a nationwide Korean cohort offers robust generalizability within this population, caution is warranted when applying these findings to other ethnicities or healthcare systems. Future studies are needed to validate these findings in diverse populations and to explore the clinical utility of serial CKM staging in relation to treatment patterns and long-term outcomes.

## 5. Conclusions

Longitudinal changes in CKM staging enhance disease monitoring and are significantly associated with adverse clinical outcomes, highlighting their importance for serial assessments to improve risk stratification, optimize preventive strategies, and mitigate long-term cardiovascular risk. Future research should investigate the causal associations and underlying mechanisms of longitudinal CKM stage changes and their impact on adverse outcomes across diverse populations and healthcare settings.

## Figures and Tables

**Table 1:** Definition of cardiovascular-kidney-metabolic syndrome stages.

Definition
Stage 0: no CKM risk factors
Individuals without overweight/obesity, metabolic risk factors (hypertension, hypertriglyceridemia, metabolic syndrome, and diabetes mellitus), CKD, or subclinical/clinical CVD.
Stage 1: excess and/or dysfunctional adiposity infarction
Individuals with overweight/obesity, abdominal obesity, or dysfunctional adipose tissue, without the presence of other metabolic risk factors or CKD. (1) BMI ≥ 23 kg/m^2^; (2) Waist circumference ≥ 85/90 cm in women/men; (3) Fasting blood glucose: 100–125 mg/dL.
Stage 2: metabolic risk factors and CKD
Individuals with metabolic risk factors (hypertriglyceridemia, hypertension, metabolic syndrome, and diabetes), or CKD. (1) Triglyceride ≥ 135 mg/dL; (2) Hypertension defined as blood pressure ≥ 140/90 mmHg or history of hypertension or use of antihypertensive drugs; (3) Metabolic syndrome defined as at least 3 of the following: -Abdominal obesity (waist circumference ≥ 85/90 cm in women/ men); -Fasting glucose ≥ 100 mg/dL or history of diabetes or use of glucose-lowering drugs; -Blood pressure ≥ 130/85 mmHg or history of hypertension or use of antihypertensive drugs; -Triglycerides ≥ 150 mg/dL; -HDL cholesterol < 40 mg/dL (men) or <50 mg/dL (women); (4) Diabetes mellitus defined as fasting glucose ≥ 126 mg/dL or history of diabetes mellitus or use of glucose-lowering drugs; (5) CKD defined as eGFR 30–59 mL/min/1.73 m^2^.
Stage 3: subclinical CVD
(1) Very-high-risk CKD defined as eGFR < 30 mL/min/1.73 m^2^; (2) High predicted 10-year CVD risk (PREVENT score ≥ 20%).
Stage 4: clinical CVD
Clinical CVD (coronary heart disease, heart failure, stroke, peripheral artery disease, and atrial fibrillation) among individuals with excess/dysfunctional adiposity, other metabolic risk factors, or CKD.

ASCVD: atherosclerotic cardiovascular disease; BMI: body mass index; CKD: chronic kidney disease; CKM: cardiovascular-kidney-metabolic syndrome; CVD: cardiovascular disease; eGFR: estimated glomerular filtration rate; HDL: high-density lipoprotein.

**Table 2:** Baseline characteristics.

Total Patients (N = 877,537)		CKM Stage Changes			*p*-Value
		Decreased (n = 96,106)	Maintained (n = 947,022)	Increased (n = 134,409)	
Age, years		45.20 ± 13.12	49.43 ± 13.91	46.04 ± 14.00	<0.001
Sex, n (%)					<0.001
	Men	56,303 (58.58)	353,242 (54.60)	81,051 (60.30)	
	Women	39,803 (41.42)	293,780 (45.40)	53,358 (39.70)	
Blood pressure, mmHg					
	SBP	118.30 ± 12.17	122.61 ± 14.79	122.66 ± 15.04	<0.001
	DBP	73.90 ± 8.35	76.41 ± 9.95	76.50 ± 9.97	<0.001
Smoking, n (%)					<0.001
	Never	54,909 (57.13)	391,275 (60.47)	74,849 (55.69)	
	Past	14,607 (15.20)	107,301 (16.58)	21,768 (16.20)	
	Current	26,590 (27.67)	148,446 (22.94)	37,792 (28.12)	
Physical activity, times/week					<0.001
	0	53,059 (55.21)	370,487 (57.26)	75,213 (55.96)	
	1–2	26,356 (27.42)	165,944 (25.65)	37,917 (28.21)	
	3–4	10,674 (11.11)	70,348 (10.87)	13,863 (10.31)	
	5–6	4350 (4.53)	27,730 (4.29)	5172 (3.85)	
	7	1667 (1.73)	12,513 (1.93)	2244 (1.67)	
Alcohol consumption, times/week					<0.001
	0	45,779 (47.63)	336,909 (52.07)	63,249 (47.06)	
	1–2	37,648 (39.17)	225,149 (34.80)	52,041 (38.72)	
	3–4	9577 (9.97)	61,983 (9.58)	14,225 (10.58)	
	≥5	3102 (3.23)	22,981 (3.55)	4894 (3.64)	
Body mass index, kg/m^2^		22.91 ± 2.93	23.92 ± 3.26	23.65 ± 2.98	<0.001
	<18.5	4458 (4.64)	21406 (3.31)	3737 (2.78)	<0.001
	18.5–22.9	51,125 (53.20)	234,807 (36.29)	50,805 (37.80)	
	23.0–24.9	19,323 (20.11)	159,768 (24.69)	40,785 (30.34)	
	≥25	21,200 (22.06)	231,041 (35.71)	39,082 (29.08)	
Waist circumference, cm		78.13 ± 8.50	80.80 ± 9.21	80.26 ± 8.61	<0.001
Fasting glucose, mg/dL		93.07 ± 16.59	97.69 ± 22.49	97.99 ± 21.28	<0.001
	<100	77,513 (80.65)	447,052 (69.09)	85,838 (63.86)	<0.001
	100–125.9	16,371 (17.03)	158,264 (24.46)	39,482 (29.37)	
	≥126	2222 (2.31)	41,706 (6.45)	9089 (6.76)	
Total cholesterol, mg/dL		187.82 ± 29.50	195.40 ± 36.99	194.66 ± 35.34	<0.001
	<200	62,391 (64.92)	374,244 (57.84)	78,704 (58.56)	<0.001
	200–239.9	32,001 (33.30)	196,324 (30.34)	39,855 (29.65)	
	≥240	1714 (1.78)	76,454 (11.82)	15,850 (11.79)	
LDL, mg/dL		111.79 ± 27.58	114.31 ± 33.62	111.77 ± 32.73	<0.001
HDL, mg/dL		56.49 ± 13.07	54.81 ± 13.50	54.79 ± 13.80	<0.001
	<40 for men or <50 for women, n (%)	13,365 (13.91)	130,261 (20.13)	26,079 (19.40)	<0.001
Triglyceride, mg/dL		97.30 ± 51.69	132.31 ± 85.12	144.19 ± 109.42	<0.001
	≥150, n (%)	5803 (6.04)	198,749 (30.72)	47,674 (35.47)	<0.001
Estimated glomerular filtration rate, mL/min/1.73 m^2^		86.78 ± 17.37	84.49 ± 18.59	79.90 ± 26.25	<0.001
Household income, quartile					<0.001
	first	21,320 (22.18)	142,689 (22.05)	28,042 (20.86)	
	second	21,201 (22.06)	130,296 (20.14)	28,830 (21.45)	
	third	26,257 (27.32)	169,047 (26.13)	36,926 (27.47)	
	fourth	27,328 (28.44)	204,990 (31.68)	40,611 (30.21)	
Hypertension, n (%)		9342 (9.72)	335,204 (51.81)	44,999 (33.48)	<0.001
Diabetes mellitus, n (%)		4069 (4.23)	72,045 (11.13)	13,189 (9.81)	<0.001
Dyslipidemia, n (%)		6883 (7.16)	191,461 (29.59)	27,153 (20.20)	<0.001
Use of antihypertensive drugs, n (%)		8443 (8.8)	284,293 (30.0)	30,164 (22.4)	
Use of glucose-lowering drugs, n (%)		3589 (3.73)	55,722 (8.61)	7551 (5.62)	<0.001
Use of lipid-lowering drugs, n (%)		6106 (6.35)	143,817 (22.23)	13,441 (10.00)	<0.001
Use of antiplatelet drugs, n (%)		10,017 (10.42)	152,463 (23.56)	20,675 (15.38)	<0.001
PREVENT score		3.33 ± 4.87	6.05 ± 8.07	6.92 ± 10.57	<0.001

CKM, cardiovascular-kidney-metabolic syndrome; DBP, diastolic blood pressure; DM, diabetes mellitus; HDL, high-density lipoprotein; LDL, low-density lipoprotein; SBP, systolic blood pressure.

**Table 3:** Clinical outcomes according to changes in CKM stage.

					Model 1			Model 2			Model 3		
	Subjects	Events	Person-year	IR	HR	95% CI	*p* value	HR	95% CI	*p* value	HR	95% CI	*p* value
Composite primary outcome (all-cause death, heart failure, stroke, or myocardial infarction)													
Decreased	96,106	6631	1,057,145	6.27	0.877	0.856–0.900	<0.001	0.875	0.853–0.897	<0.001	0.997	0.972–1.024	0.849
Maintained	647,022	69,728	6,949,014	10.03	1 (Ref.)			1 (Ref.)			1 (Ref.)		
Increased	134,409	12,307	1,461,135	8.42	1.031	1.012–1.051	0.002	1.014	0.995–1.034	0.153	1.071	1.050–1.092	<0.001
All-cause death													
Decreased	96,106	3568	1,072,485	3.33	0.977	0.943–1.011	0.179	0.927	0.895–0.959	<0.001	1.028	0.992–1.065	0.124
Maintained	647,022	37,056	7,127,035	5.20	1 (Ref.)			1 (Ref.)			1 (Ref.)		
Increased	134,409	6470	1,493,786	4.33	1.032	1.005–1.060	0.018	1.009	0.983–1.036	0.512	1.043	1.015–1.071	0.002
Heart failure													
Decreased	96,106	2170	1,065,497	2.04	0.762	0.729–0.796	<0.001	0.789	0.755–0.824	<0.001	0.966	0.924–1.010	0.127
Maintained	647,022	26,777	7,036,946	3.81	1 (Ref.)			1 (Ref.)			1 (Ref.)		
Increased	134,409	4724	1,477,392	3.20	1.046	1.014–1.079	0.005	1.038	1.007–1.071	0.017	1.131	1.096–1.166	<0.001
Stroke (ischemic or hemorrhagic)													
Decreased	96,106	1899	1,064,757	1.78	0.869	0.829–0.911	<0.001	0.886	0.845–0.929	<0.001	1.012	0.965–1.062	0.617
Maintained	647,022	20,202	7,044,115	2.87	1 (Ref.)			1 (Ref.)			1 (Ref.)		
Increased	134,409	3675	1,478,291	2.49	1.06	1.023–1.098	0.001	1.047	1.011–1.085	0.011	1.106	1.068–1.146	<0.001
Myocardial infarction													
Decreased	96,106	531	1,070,110	0.50	0.792	0.724–0.866	<0.001	0.838	0.766–0.916	<0.001	0.948	0.866–1.039	0.252
Maintained	647,022	5531	7,102,163	0.78	1 (Ref.)			1 (Ref.)			1 (Ref.)		
Increased	134,409	960	1,489,420	0.64	0.928	0.867–0.994	0.033	0.921	0.859–0.986	0.018	0.979	0.913–1.049	0.541

CI, confidence interval; HR, hazard ratio; IR, incidence rate; MI, myocardial infarction; and Ref, reference. Model 1: adjusted by age and sex. Model 2: adjusted for age, sex, smoking status, alcohol consumption, physical activity, and household income. Model 3: Adjusted for age, sex, smoking status, alcohol consumption, physical activity, household income, use of antihypertensive drugs, glucose-lowering drugs, lipid-lowering drugs, and antiplatelet drugs.

**Table 4:** Subgroup analysis of composite primary outcome according to changes in CKM stage by sex and age groups.

					Model 1			Model 2			Model 3		
	Subjects	Events	Person-year	IR	HR	95% CI	*p* value	HR	95% CI	*p* value	HR	95% CI	*p* value
Composite primary outcome according to sex, interaction *p*-value: <0.001													
Men													
Decreased	56,303	4392	619,251	7.09	0.900	0.872–0.928	<0.001	0.883	0.856–0.911	<0.001	0.990	0.959–1.022	0.521
Maintained	353,242	42,173	3,792,676	11.12	1 (Ref.)			1 (Ref.)			1 (Ref.)		
Increased	81,051	8028	882,481	9.10	1.017	0.993–1.042	0.155	0.992	0.968–1.016	0.497	1.040	1.016–1.066	0.001
Women													
Decreased	39,803	2239	437,894	5.11	0.832	0.797–0.869	<0.001	0.848	0.812–0.885	<0.001	0.994	0.951–1.039	0.799
Maintained	293,780	27,555	3,156,337	8.73	1 (Ref.)			1 (Ref.)			1 (Ref.)		
Increased	53,358	4279	578,654	7.39	1.056	1.023–1.091	<0.001	1.054	1.02–1.088	0.002	1.124	1.088–1.161	<0.001
Composite Primary Outcome According to Age Groups, Interaction *p*-Value: <0.001													
20–29 Years													
Decreased	15,572	154	175,172	0.88	0.857	0.721–1.019	0.081	0.896	0.754–1.065	0.214	0.987	0.829–1.176	0.886
Maintained	71,974	792	809,500	0.98	1 (Ref.)			1 (Ref.)			1 (Ref.)		
Increased	22,381	231	253,233	0.91	0.86	0.742–0.996	0.044	0.854	0.737–0.989	0.035	0.906	0.781–1.050	0.189
30–39 Years													
Decreased	23,728	409	268,668	1.52	0.765	0.689–0.849	<0.001	0.81	0.730–0.900	<0.001	0.904	0.813–1.006	0.064
Maintained	117,384	2667	1,322,499	2.02	1 (Ref.)			1 (Ref.)			1 (Ref.)		
Increased	30,890	598	350,511	1.71	0.855	0.783–0.935	<0.001	0.884	0.809–0.967	0.007	0.958	0.875–1.048	0.345
40–49 Years													
Decreased	26,458	956	294,455	3.25	0.79	0.738–0.845	<0.001	0.821	0.767–0.878	<0.001	0.981	0.916–1.052	0.595
Maintained	162,841	7668	1,802,825	4.25	1 (Ref.)			1 (Ref.)			1 (Ref.)		
Increased	35,866	1469	399,512	3.68	0.89	0.842–0.941	<0.001	0.907	0.858–0.959	<0.001	1.03	0.973–1.090	0.313
50–59 years													
Decreased	16,949	1405	184,740	7.61	0.859	0.813–0.908	<0.001	0.859	0.813–0.908	<0.001	1.044	0.987–1.105	0.134
Maintained	153,472	14,629	1,655,223	8.84	1 (Ref.)			1 (Ref.)			1 (Ref.)		
Increased	22,886	2,232	247,439	9.02	1.017	0.972–1.063	0.466	1.01	0.966–1.056	0.655	1.136	1.085–1.188	<0.001
≥60 years													
Decreased	13,399	3707	134,111	27.64	0.923	0.892–0.954	<0.001	0.910	0.880–0.942	<0.001	1.025	0.991–1.061	0.156
Maintained	141,351	43,972	1,358,967	32.36	1 (Ref.)			1 (Ref.)			1 (Ref.)		
Increased	22,386	7777	210,441	36.96	1.082	1.056–1.109	<0.001	1.062	1.037–1.088	<0.001	1.096	1.070–1.123	<0.001

CI, confidence interval; HR, hazard ratio; IR, incidence rate; MI, myocardial infarction; and Ref, reference. Model 1: adjusted by age and sex. Model 2: adjusted for age, sex, smoking status, alcohol consumption, physical activity, and household income. Model 3: Adjusted for age, sex, smoking status, alcohol consumption, physical activity, household income, use of antihypertensive drugs, glucose-lowering drugs, lipid-lowering drugs, and antiplatelet drugs.

## Data Availability

This study used data from the NHID of Korea. The data are not publicly available but can be accessed through the NHID data request process for approved research purposes (https://nhiss.nhis.or.kr (accessed on 6 November 2024)).

## Supplementary Materials

The following supporting information can be downloaded at: https://www.mdpi.com/article/10.3390/jcm14113888/s1 Figure S1: CKM stage flow between health check-ups by sex and age subgroups; Figure S2: Kaplan–Meier curve for clinical outcomes according to changes in CKM stage; Table S1: diagnoses and procedural definitions of comorbidities and clinical outcomes based on ICD-10 codes; Table S2: changes in CKM stages between first and second health check-ups; Table S3: changes in CKM stages by sex between first and second health check-ups; Table S4: changes in CKM stages by age group between first and second health check-ups; Table S5: clinical outcomes according to changes in CKM stage in men; Table S6: clinical outcomes according to changes in CKM stage in women; Table S7: clinical outcomes according to changes in CKM stage in patients aged 20–29 years; Table S8: clinical outcomes according to changes in CKM stage in patients aged 30–39 years; Table S9: clinical outcomes according to changes in CKM stage in patients aged 40–49 years; Table S10: clinical outcomes according to changes in CKM stage in patients aged 50–59 years; Table S11: clinical outcomes according to changes in CKM stage in patients aged ≥ 60 years; Table S12: clinical outcomes according to changes in CKM stage among participants initially at stage 0; Table S13: clinical outcomes according to changes in CKM stage among participants initially at stage 1; Table S14: clinical outcomes according to changes in CKM stage among participants initially at stage 2; Table S15: clinical outcomes according to changes in CKM stage among participants initially at stage 3.

## Abbreviations

The following abbreviations are used in this manuscript:

ASCVD	Atherosclerotic cardiovascular disease
AHA	American Heart Association
BMI	Body mass index
BP	Blood pressure
CKD	Chronic kidney disease
CKM	Cardiovascular-kidney-metabolic
CI	Confidence interval
CVD	Cardiovascular disease
DM	Diabetes mellitus
eGFR	Estimated glomerular filtration rate
HR	Hazard ratio
ICD-10	*International Classification of Diseases, 10th Revision*
MI	Myocardial infarction
NHID	National Health Insurance Database
PREVENT	Predicting risk of cardiovascular disease events
SAS	Statistical analysis system
SBP	Systolic blood pressure
