# Supplementary material for: Longitudinal Changes in Cardiovascular-Kidney-Metabolic Syndrome Stages and Their Impact on Outcomes: A Nationwide Cohort Study
Source: J Clin Med. 2025 Jun 1;14(11):3888. doi: 10.3390/jcm14113888 (PMC12156345; doi:10.3390/jcm14113888)
Supplement: Supplementary file 1 [file jcm-14-03888-s001.zip › jcm-3650025-supplementary.pdf]

Supplementary material

Table S1. Diagnoses and procedural definitions of comorbidities and clinical outcomes based on ICD-10 codes

Table S2. Changes in CKM stages between first and second health check-ups

Table S3. Changes in CKM stages by sex between first and second health check-ups

Table S4. Changes in CKM stages by age group between first and second health check-ups

Table S5. Clinical outcomes according to changes in CKM stage in men

Table S6. Clinical outcomes according to changes in CKM stage in women

Table S7. Clinical outcomes according to changes in CKM stage in patients aged 20-29 years

Table S8. Clinical outcomes according to changes in CKM stage in patients aged 30-39 years

Table S9. Clinical outcomes according to changes in CKM stage in patients aged 40-49 years

Table S10. Clinical outcomes according to changes in CKM stage in patients aged 50-59 years

Table S11. Clinical outcomes according to changes in CKM stage in patients aged ≥60 years

Table S12. Clinical outcomes according to changes in CKM stage among participants initially at stage 0

Table S13. Clinical outcomes according to changes in CKM stage among participants initially at stage 1

Table S14. Clinical outcomes according to changes in CKM stage among participants initially at stage 2

Table S15. Clinical outcomes according to changes in CKM stage among participants initially at stage 3

Figure S1. CKM stage flow between health check-ups by sex and age subgroups.

(A) Men. (B) Women. (C) 20-29 years. (D) 30-39 years. (E) 40-49 years. (F) 50-59 years. (G)

≥60 years.

Figure S2. Kaplan-Meier curve for clinical outcomes according to changes in CKM stage. (A) Composite primary outcome (all-cause death, heart failure, stroke, or myocardial infarction). (B) All-cause death. (C) Heart failure. (D) Stroke (ischemic or hemorrhagic). (E) Myocardial infarction.

Table S1. Diagnoses and procedural definitions of comorbidities and clinical outcomes based on ICD-10 codes

Diagnosis	Definition
Hypertension	Defined as blood pressure ≥140/90 mmHg, a history of hypertension (ICD-10 codes I10–I15), or the use of antihypertensive drugs.
Diabetes mellitus	Defined as fasting glucose ≥126 mg/dL, a history of diabetes mellitus (ICD-10 codes E10–E14), or the use of glucose-lowering drugs.
Dyslipidemia	Defined as total cholesterol ≥240 mg/dL, a history of dyslipidemia (ICD-10 code E78), or the use of lipid-lowering drugs.
Heart failure	Defined as hospital admission with ICD-10 codes I50, I42.0, I11.0, or I13.0–I13.2 as primary diagnosis.
Myocardial infarction	Defined as hospital admission with ICD-10 codes I21-I22 as primary diagnosis and who had undergone coronary revascularization, including PCI or CABG, based on procedure codes.
Coronary artery disease	Defined as a history of myocardial infarction or coronary revascularization.
Coronary artery bypass graft surgery	
	Defined as procedure codes O1640–O1642, O1647–O1649, OA640–OA642, or OA647–OA649
Percutaneous coronary intervention	
	Defined as procedure codes M6551–M6554, M6561–M6567, M6571, M6572, or O1876–O1877
Stroke	Defined as hospital admission with ICD-10 codes I63–I64 (ischemic stroke) or with ICD-10 codes I60–I62 (hemorrhagic stroke) as the primary diagnosis, accompanied by a brain imaging procedure code
Brain imaging	
	Defined as procedure codes HA441, HA451, HA461, HA851, HE101, HE201, HE301, HE401, HE501, HE102, HE135, HE136, HE202, HE235, HE236, HE301, HE302, HE501, HE502, HE535, HE536, HI101, HI135, HI136, HI201, HI235, HI236, HI301, HI401, HI501, HI535, HI536, HJ101, HJ135, HJ136, HJ201, HJ235, HJ236, HJ301, HJ401, HJ501, HJ535, or HJ536
Peripheral artery disease	Defined as hospital admission with ICD-10 codes I70– 71, I73.1, I73.8–9, I77.1, I79.0, I79.2, K55.1, K55.8–9, Z95.8–9 as primary diagnosis, along with evidence of peripheral artery revascularization procedures.
Peripheral artery revascularization	
	Defined as procedure codes M6597, M6605, M6620, M6613, M6632, O0161~70, O1645, O1646, O2064, O2065, O2067, O2068
Atrial fibrillation	Defined as ICD-10 code I48.

CABG, coronary artery bypass grafting; ICD, International Classification of Diseases; PCI, percutaneous coronary intervention.

Table S2. Changes in CKM stages between first and second health check-ups

**Total patients** (n=877,537)		**CKM stages at 1st health check-up**				
		**Stage 0** (n=158,409)	**Stage 1** (n=143,914)	**Stage 2** (n=502,033)	**Stage 3** (n=48,246)	**Stage 4** (n=24,935)
**CKM stages at 2nd health check-up**	**Stage 0**	102402 (64.64)	19816 (13.77)	25591 (5.10)	881 (1.83)	0 (0.00)
	**Stage 1**	22292 (14.07)	71515 (49.69)	37640 (7.50)	770 (1.60)	0 (0.00)
	**Stage 2**	31147 (19.66)	49952 (34.71)	414351 (82.53)	11408 (23.65)	0 (0.00)
	**Stage 3**	2381 (1.50)	2340 (1.63)	20604 (4.10)	33819 (70.10)	0 (0.00)
	**Stage 4**	187 (0.12)	291 (0.20)	3847 (0.77)	1368 (2.84)	24935 (100.00)

CKM: Cardiovascular-Kidney-Metabolic syndrome

Table S3. Changes in CKM stages by sex between first and second health check-ups

**Men** (n=490,596)		CKM stages at 1st health check-up				
		**Stage 0** (n=64,602)	**Stage 1** (n=86,693)	**Stage 2** (n=296,251)	**Stage 3** (n=28,665)	**Stage 4** (n=14,385)
**CKM stages at 2nd health check-up**	**Stage 0**	35860 (55.51)	9263 (10.68)	12779 (4.31)	353 (1.23)	0 (0.00)
	**Stage 1**	11206 (17.35)	42443 (48.96)	26016 (8.78)	516 (1.80)	0 (0.00)
	**Stage 2**	16169 (25.03)	32990 (38.05)	241004 (81.35)	7376 (25.73)	0 (0.00)
	**Stage 3**	1267 (1.96)	1805 (2.08)	14130 (4.77)	19550 (68.20)	0 (0.00)
	**Stage 4**	100 (0.15)	192 (0.22)	2322 (0.78)	870 (3.04)	14385 (100.00)
**Women** (n=386,941)		**CKM stages at 1st health check-up**				
		**Stage 0** (n=93,807)	**Stage 1** (n=57,221)	**Stage 2** (n=205,782)	**Stage 3** (n=19,581)	**Stage 4** (n=10,550)
**CKM stages at 2nd health check-up**	**Stage 0**	66542 (70.94)	10553 (18.44)	12812 (6.23)	528 (2.70)	0 (0.00)
	**Stage 1**	11086 (11.82)	29072 (50.81)	11624 (5.65)	254 (1.30)	0 (0.00)
	**Stage 2**	14978 (15.97)	16962 (29.64)	173347 (84.24)	4032 (20.59)	0 (0.00)
	**Stage 3**	1114 (1.19)	535 (0.93)	6474 (3.15)	14269 (72.87)	0 (0.00)
	**Stage 4**	87 (0.09)	99 (0.17)	1525 (0.74)	498 (2.54)	10550 (100.00)

CKM: Cardiovascular-Kidney-Metabolic syndrome

Table S4. Changes in CKM stages by age group between first and second health check-ups

**20-29 years** (n=109,927)		**CKM stages at 1st health check-up**				
		**Stage 0** (n=49,756)	**Stage 1** (n=27,139)	**Stage 2** (n=31,396)	**Stage 3** (n=1,302)	**Stage 4** (n=334)
**CKM stages at 2nd health check-up**	**Stage 0**	35904 (72.16)	4998 (18.42)	4320 (13.76)	456 (35.02)	0 (0.00)
	**Stage 1**	6298 (12.66)	14368 (52.94)	5082 (16.19)	306 (23.50)	0 (0.00)
	**Stage 2**	6487 (13.04)	7155 (26.36)	21240 (67.65)	410 (31.49)	0 (0.00)
	**Stage 3**	1043 (2.10)	602 (2.22)	716 (2.28)	128 (9.83)	0 (0.00)
	**Stage 4**	24 (0.05)	16 (0.06)	38 (0.12)	2 (0.15)	334 (100.00)
**30-39 years** (n=172,002)		**CKM stages at 1st health check-up**				
		**Stage 0** (n=44,751)	**Stage 1** (n=39,318)	**Stage 2** (n=85,414)	**Stage 3** (n=1,512)	**Stage 4** (n=1,007)
**CKM stages at 2nd health check-up**	**Stage 0**	29600 (66.14)	5579 (14.19)	6452 (7.55)	136 (8.99)	0 (0.00)
	**Stage 1**	6298 (14.07)	20034 (50.95)	10544 (12.34)	176 (11.64)	0 (0.00)
	**Stage 2**	8224 (18.38)	13055 (33.20)	66392 (77.73)	841 (55.62)	0 (0.00)
	**Stage 3**	588 (1.31)	610 (1.55)	1884 (2.21)	351 (23.21)	0 (0.00)
	**Stage 4**	41 (0.09)	40 (0.10)	142 (0.17)	8 (0.53)	1007 (100.00)
**40-49 years** (n=225,165)		**CKM stages at 1st health check-up**				
		**Stage 0** (n=42,051)	**Stage 1** (n=45,405)	**Stage 2** (n=132,489)	**Stage 3** (n=2,333)	**Stage 4** (n=2,887)
**CKM stages at 2nd health check-up**	**Stage 0**	25848 (61.47)	5929 (13.06)	7479 (5.64)	104 (4.46)	0 (0.00)
	**Stage 1**	6413 (15.25)	22941 (50.53)	11423 (8.62)	102 (4.37)	0 (0.00)
	**Stage 2**	9420 (22.40)	16031 (35.31)	110481 (83.39)	1421 (60.91)	0 (0.00)
	**Stage 3**	316 (0.75)	421 (0.93)	2529 (1.91)	684 (29.32)	0 (0.00)
	**Stage 4**	54 (0.13)	83 (0.18)	577 (0.44)	22 (0.94)	2887 (100.00)
**50-59 years**		**CKM stages at 1st health check-up**				

(n=193,307)		Stage 0 (n=16,642)	Stage 1 (n=23,652)	Stage 2 (n=143,313)	Stage 3 (n=3,628)	Stage 4 (n=6,072)
**CKM stages at 2nd health check-up**	**Stage 0**	8716 (52.37)	2501 (10.57)	4869 (3.40)	41 (1.13)	0 (0.00)
	**Stage 1**	2510 (15.08)	10833 (45.80)	7303 (5.10)	72 (1.98)	0 (0.00)
	**Stage 2**	5219 (31.36)	9906 (41.88)	126575 (88.32)	2163 (59.62)	0 (0.00)
	**Stage 3**	158 (0.95)	319 (1.35)	3363 (2.35)	1276 (35.17)	0 (0.00)
	**Stage 4**	39 (0.23)	93 (0.39)	1203 (0.84)	76 (2.09)	6072 (100.00)
**≥60 years** (n=177,136)		**CKM stages at 1st health check-up**				
		**Stage 0** (n=5,209)	**Stage 1** (n=8,400)	**Stage 2** (n=109,421)	**Stage 3** (n=39,471)	**Stage 4** (n=14,635)
**CKM stages at 2nd health check-up**	**Stage 0**	2334 (44.81)	809 (9.63)	2471 (2.26)	144 (0.36)	0 (0.00)
	**Stage 1**	773 (14.84)	3339 (39.75)	3288 (3.00)	114 (0.29)	0 (0.00)
	**Stage 2**	1797 (34.50)	3805 (45.30)	89663 (81.94)	6573 (16.65)	0 (0.00)
	**Stage 3**	276 (5.30)	388 (4.62)	12112 (11.07)	31380 (79.50)	0 (0.00)
	**Stage 4**	29 (0.56)	59 (0.70)	1887 (1.72)	1260 (3.19)	14635 (100.00)

CKM: Cardiovascular-Kidney-Metabolic syndrome

Table S5. Clinical outcomes according to changes in CKM stage in men

					Model 1			Model 2			Model 3		
	Subjects	Events	Person-year	IR	HR	95% CI	p value	HR	95% CI	p value	HR	95% CI	p value
**All-cause death**													
Decreased	56303	2499	628949	3.97	0.998	0.957-1.040	0.916	0.932	0.894-0.972	0.001	1.032	0.989-1.077	0.143
Maintained	353242	24071	3893244	6.18	1 (Ref.)			1 (Ref.)			1 (Ref.)		
Increased	81051	4484	902819	4.97	1.022	0.990-1.055	0.186	0.988	0.956-1.020	0.442	1.023	0.990-1.056	0.171
**Heart failure**													
Decreased	56303	1253	624990	2.00	0.766	0.723-0.812	<.001	0.781	0.737-0.828	<.001	0.936	0.883-0.993	0.029
Maintained	353242	14220	3847145	3.70	1 (Ref.)			1 (Ref.)			1 (Ref.)		
Increased	81051	2763	893623	3.09	1.034	0.993-1.077	0.105	1.022	0.981-1.065	0.303	1.1	1.056-1.145	<.001
**Stroke (ischemic or hemorrhagic)**													
Decreased	56303	1185	624084	1.90	0.876	0.825-0.930	<.001	0.885	0.834-0.940	<.001	0.994	0.935-1.056	0.835
Maintained	353242	11671	3845892	3.03	1 (Ref.)			1 (Ref.)			1 (Ref.)		
Increased	81051	2333	892852	2.61	1.061	1.015-1.110	0.009	1.043	0.997-1.090	0.066	1.095	1.047-1.145	<.001

**Myocardial infarction**													
Decreased	56303	444	626925	0.71	0.801	0.726-0.883	<.001	0.848	0.769-0.936	0.001	0.945	0.856-1.045	0.271
Maintained	353242	4291	3873622	1.11	1 (Ref.)			1 (Ref.)			1 (Ref.)		
Increased	81051	773	899192	0.86	0.905	0.838-0.977	0.011	0.901	0.835-0.973	0.008	0.952	0.881-1.028	0.208

CKM, Cardiovascular-Kidney-Metabolic syndrome; CI, confidence interval; HR, hazard ratio; IR, incidence rate; MI, myocardial infarction

Model 1: adjusted by age, sex.

Model 2: adjusted for age, sex, smoking status, alcohol consumption, physical activity, and household income.

Model 3: Adjusted for age, sex, smoking status, alcohol consumption, physical activity, household income, use of antihypertensive drugs, glucose-lowering drugs, lipid-lowering drugs, and antiplatelet drugs.

Table S6. Clinical outcomes according to changes in CKM stage in women

	Subjects	Events	Person-year	IR	Model 1			Model 2			Model 3		
					HR	95% CI	p value	HR	95% CI	p value	HR	95% CI	p value
**All-cause death**													
Decreased	39803	1069	443536	2.41	0.921	0.865-0.980	0.010	0.893	0.838-0.950	<.001	0.992	0.931-1.058	0.816
Maintained	293780	12985	3233790	4.02	1 (Ref.)			1 (Ref.)			1 (Ref.)		
Increased	53358	1986	590967	3.36	1.051	1.003-1.102	0.039	1.052	1.003-1.103	0.037	1.079	1.029-1.131	0.002
**Heart failure**													
Decreased	39803	917	440508	2.08	0.752	0.703-0.804	<.001	0.793	0.741-0.848	<.001	0.993	0.928-1.064	0.850
Maintained	293780	12557	3189801	3.94	1 (Ref.)			1 (Ref.)			1 (Ref.)		
Increased	53358	1961	583769	3.36	1.06	1.010-1.111	0.017	1.06	1.010-1.111	0.017	1.168	1.113-1.226	<.001
**Stroke (ischemic or hemorrhagic)**													
Decreased	39803	714	440673	1.62	0.855	0.792-0.923	<.001	0.883	0.818-0.954	0.002	1.031	0.953-1.115	0.451
Maintained	293780	8531	3198223	2.67	1 (Ref.)			1 (Ref.)			1 (Ref.)		
Increased	53358	1342	585439	2.29	1.054	0.995-1.117	0.071	1.053	0.994-1.116	0.077	1.12	1.057-1.187	<.001

**Myocardial infarction**													
Decreased	39803	87	443185	0.20	0.736	0.592-0.916	0.006	0.766	0.615-0.953	0.017	0.927	0.743-1.157	0.502
Maintained	293780	1240	3228541	0.38	1 (Ref.)			1 (Ref.)			1 (Ref.)		
Increased	53358	187	590228	0.32	1.014	0.869-1.183	0.860	1.013	0.868-1.181	0.871	1.091	0.935-1.274	0.270

CKM, Cardiovascular-Kidney-Metabolic syndrome; CI, confidence interval; HR, hazard ratio; IR, incidence rate; MI, myocardial infarction

Model 1: adjusted by age, sex.

Model 2: adjusted for age, sex, smoking status, alcohol consumption, physical activity, and household income.

Model 3: Adjusted for age, sex, smoking status, alcohol consumption, physical activity, household income, use of antihypertensive drugs, glucose-lowering drugs, lipid-lowering drugs, and antiplatelet drugs.

Table S7. Clinical outcomes according to changes in CKM stage in patients aged 20-29 years

					Model 1			Model 2			Model 3		
	Subjects	Events	Person-year	IR	HR	95% CI	p value	HR	95% CI	p value	HR	95% CI	p value
**All-cause death**													
Decreased	15572	70	175509	0.40	0.910	0.703-1.177	0.472	0.939	0.725-1.216	0.633	0.969	0.747-1.257	0.812
Maintained	71974	336	811186	0.41	1 (Ref.)			1 (Ref.)			1 (Ref.)		
Increased	22381	96	253690	0.38	0.831	0.662-1.044	0.112	0.830	0.661-1.042	0.108	0.845	0.673-1.062	0.150
**Heart failure**													
Decreased	15572	50	175332	0.29	0.786	0.582-1.062	0.117	0.828	0.613-1.119	0.219	0.972	0.716-1.320	0.855
Maintained	71974	285	810246	0.35	1 (Ref.)			1 (Ref.)			1 (Ref.)		
Increased	22381	73	253492	0.29	0.774	0.598-1.002	0.052	0.765	0.591-0.989	0.041	0.839	0.647-1.088	0.185
**Stroke (ischemic or hemorrhagic)**													
Decreased	15572	38	175355	0.22	1.023	0.719-1.456	0.899	1.093	0.768-1.557	0.621	1.248	0.871-1.788	0.227
Maintained	71974	166	810490	0.20	1 (Ref.)			1 (Ref.)			1 (Ref.)		
Increased	22381	57	253471	0.22	1.037	0.767-1.403	0.814	1.021	0.755-1.381	0.892	1.111	0.820-1.506	0.497

**Myocardial infarction**													
Decreased	15572	11	175480	0.06	0.88	0.459-1.689	0.701	0.98	0.509-1.886	0.952	1.124	0.578-2.188	0.730
Maintained	71974	51	811031	0.06	1 (Ref.)			1 (Ref.)			1 (Ref.)		
Increased	22381	12	253643	0.05	0.611	0.326-1.146	0.125	0.626	0.334-1.176	0.145	0.69	0.365-1.301	0.251

CKM, Cardiovascular-Kidney-Metabolic syndrome; CI, confidence interval; HR, hazard ratio; IR, incidence rate; MI, myocardial infarction

Model 1: adjusted by age, sex.

Model 2: adjusted for age, sex, smoking status, alcohol consumption, physical activity, and household income.

Model 3: Adjusted for age, sex, smoking status, alcohol consumption, physical activity, household income, use of antihypertensive drugs, glucose-lowering drugs, lipid-lowering drugs, and antiplatelet drugs.

Table S8. Clinical outcomes according to changes in CKM stage in patients aged 30-39 years

	Subjects	Events	Person-year	IR	Model 1			Model 2			Model 3		
					HR	95% CI	p value	HR	95% CI	p value	HR	95% CI	p value
**All-cause death**													
Decreased	23728	166	269640	0.62	0.811	0.688-0.955	0.012	0.812	0.688-0.958	0.013	0.875	0.740-1.035	0.119
Maintained	117384	1022	1329465	0.77	1 (Ref.)			1 (Ref.)			1 (Ref.)		
Increased	30890	231	352115	0.66	0.862	0.748-0.995	0.042	0.866	0.750-0.999	0.049	0.913	0.791-1.055	0.220
**Heart failure**													
Decreased	23728	138	269238	0.51	0.793	0.663-0.949	0.011	0.849	0.708-1.017	0.075	0.980	0.815-1.178	0.828
Maintained	117384	866	1326583	0.65	1 (Ref.)			1 (Ref.)			1 (Ref.)		
Increased	30890	199	351460	0.57	0.874	0.749-1.020	0.088	0.903	0.773-1.054	0.194	1.002	0.857-1.172	0.976
**Stroke (ischemic or hemorrhagic)**													
Decreased	23728	113	269131	0.42	0.814	0.667-0.993	0.042	0.896	0.733-1.094	0.281	1.011	0.825-1.240	0.915
Maintained	117384	696	1326453	0.52	1 (Ref.)			1 (Ref.)			1 (Ref.)		
Increased	30890	151	351427	0.43	0.832	0.698-0.992	0.041	0.876	0.734-1.045	0.141	0.953	0.797-1.139	0.595

**Myocardial infarction**													
Decreased	23728	33	269521	0.12	0.44	0.308-0.628	<.001	0.514	0.359-0.735	<.001	0.565	0.394-0.811	0.002
Maintained	117384	378	1327832	0.28	1 (Ref.)			1 (Ref.)			1 (Ref.)		
Increased	30890	87	351744	0.25	0.88	0.697-1.112	0.285	0.99	0.783-1.252	0.934	1.068	0.843-1.354	0.585

CKM, Cardiovascular-Kidney-Metabolic syndrome; CI, confidence interval; HR, hazard ratio; IR, incidence rate; MI, myocardial infarction

Model 1: adjusted by age, sex.

Model 2: adjusted for age, sex, smoking status, alcohol consumption, physical activity, and household income.

Model 3: Adjusted for age, sex, smoking status, alcohol consumption, physical activity, household income, use of antihypertensive drugs, glucose-lowering drugs, lipid-lowering drugs, and antiplatelet drugs.

Table S9. Clinical outcomes according to changes in CKM stage in patients aged 40-49 years

	Subjects	Events	Person-year	IR	Model 1			Model 2			Model 3		
					HR	95% CI	p value	HR	95% CI	p value	HR	95% CI	p value
**All-cause death**													
Decreased	26458	402	296878	1.35	0.926	0.834-1.028	0.148	0.898	0.808-0.998	0.045	1.02	0.916-1.137	0.715
Maintained	162841	2777	1825488	1.52	1 (Ref.)			1 (Ref.)			1 (Ref.)		
Increased	35866	544	403765	1.35	0.917	0.836-1.005	0.063	0.904	0.824-0.991	0.031	0.99	0.902-1.088	0.841
**Heart failure**													
Decreased	26458	289	295839	0.98	0.702	0.622-0.793	<.001	0.764	0.676-0.863	<.001	0.989	0.872-1.121	0.862
Maintained	162841	2603	1816123	1.43	1 (Ref.)			1 (Ref.)			1 (Ref.)		
Increased	35866	508	401935	1.26	0.901	0.819-0.991	0.032	0.939	0.854-1.033	0.199	1.126	1.022-1.241	0.017
**Stroke (ischemic or hemorrhagic)**													
Decreased	26458	266	295740	0.90	0.717	0.631-0.814	<.001	0.753	0.662-0.855	<.001	0.902	0.791-1.028	0.121
Maintained	162841	2347	1814928	1.29	1 (Ref.)			1 (Ref.)			1 (Ref.)		
Increased	35866	446	401804	1.11	0.880	0.795-0.974	0.013	0.9	0.813-0.996	0.041	1.02	0.920-1.131	0.707

**Myocardial infarction**														
Decreased	26458	106	296395	0.36	0.667	0.546-0.815	<.001	0.741	0.606-0.906	0.004	0.878	0.716-1.078	0.214
Maintained	162841	1035	1820649	0.57	1 (Ref.)			1 (Ref.)			1 (Ref.)		
Increased	35866	165	402966	0.41	0.762	0.646-0.898	0.001	0.81	0.687-0.955	0.012	0.907	0.768-1.072	0.253

CKM, Cardiovascular-Kidney-Metabolic syndrome; CI, confidence interval; HR, hazard ratio; IR, incidence rate; MI, myocardial infarction

Model 1: adjusted by age, sex.

Model 2: adjusted for age, sex, smoking status, alcohol consumption, physical activity, and household income.

Model 3: Adjusted for age, sex, smoking status, alcohol consumption, physical activity, household income, use of antihypertensive drugs, glucose-lowering drugs, lipid-lowering drugs, and antiplatelet drugs.

Table S10. Clinical outcomes according to changes in CKM stage in patients aged 50-59 years

	Subjects	Events	Person-year	IR	Model 1			Model 2			Model 3		
					HR	95% CI	p value	HR	95% CI	p value	HR	95% CI	p value
**All-cause death**													
Decreased	16949	628	188261	3.34	0.986	0.908-1.071	0.742	0.918	0.845-0.998	0.044	1.047	0.961-1.141	0.290
Maintained	153472	5686	1699046	3.35	1 (Ref.)			1 (Ref.)			1 (Ref.)		
Increased	22886	980	254159	3.86	1.129	1.055-1.208	<.001	1.086	1.015-1.163	0.017	1.164	1.086-1.247	<.001
**Heart failure**													
Decreased	16949	460	186842	2.46	0.759	0.690-0.835	<.001	0.802	0.729-0.883	<.001	1.094	0.991-1.208	0.074
Maintained	153472	5524	1678688	3.29	1 (Ref.)			1 (Ref.)			1 (Ref.)		
Increased	22886	789	251089	3.14	0.972	0.902-1.048	0.463	0.992	0.921-1.069	0.839	1.195	1.107-1.289	<.001
**Stroke (ischemic or hemorrhagic)**													
Decreased	16949	415	186467	2.23	0.823	0.744-0.910	<.001	0.834	0.753-0.922	<.001	0.992	0.894-1.101	0.877
Maintained	153472	4546	1678573	2.71	1 (Ref.)			1 (Ref.)			1 (Ref.)		
Increased	22886	693	250976	2.76	1.019	0.940-1.103	0.653	1.017	0.938-1.102	0.683	1.126	1.037-1.221	0.005

**Myocardial infarction**													
Decreased	16949	146	187561	0.78	0.844	0.712-1.001	0.051	0.862	0.727-1.023	0.089	1.043	0.876-1.243	0.634
Maintained	153472	1508	1691785	0.89	1 (Ref.)			1 (Ref.)			1 (Ref.)		
Increased	22886	255	252906	1.01	1.074	0.940-1.227	0.292	1.071	0.937-1.223	0.315	1.187	1.037-1.358	0.013

CKM, Cardiovascular-Kidney-Metabolic syndrome; CI, confidence interval; HR, hazard ratio; IR, incidence rate; MI, myocardial infarction

Model 1: adjusted by age, sex.

Model 2: adjusted for age, sex, smoking status, alcohol consumption, physical activity, and household income.

Model 3: Adjusted for age, sex, smoking status, alcohol consumption, physical activity, household income, use of antihypertensive drugs, glucose-lowering drugs, lipid-lowering drugs, and antiplatelet drugs.

Table S11. Clinical outcomes according to changes in CKM stage in patients aged ≥60 years

	Subjects	Events	Person-year	IR	Model 1			Model 2			Model 3		
					HR	95% CI	p value	HR	95% CI	p value	HR	95% CI	p value
**All-cause death**													
Decreased	13399	2302	142197	16.19	1.013	0.971-1.057	0.550	0.965	0.925-1.008	0.109	1.083	1.037-1.131	<.001
Maintained	141351	27235	1461849	18.63	1 (Ref.)			1 (Ref.)			1 (Ref.)		
Increased	22386	4619	230057	20.08	1.042	1.010-1.075	0.010	1.028	0.996-1.060	0.088	1.057	1.024-1.091	<.001
**Heart failure**													
Decreased	13399	1233	138246	8.92	0.768	0.724-0.813	<.001	0.784	0.740-0.831	<.001	0.937	0.884-0.994	0.031
Maintained	141351	17499	1405306	12.45	1 (Ref.)			1 (Ref.)			1 (Ref.)		
Increased	22386	3155	219416	14.38	1.111	1.069-1.154	<.001	1.091	1.050-1.133	<.001	1.144	1.101-1.188	<.001
**Stroke (ischemic or hemorrhagic)**													
Decreased	13399	1067	138063	7.73	0.926	0.870-0.986	0.017	0.933	0.876-0.993	0.030	1.045	0.981-1.114	0.174
Maintained	141351	12447	1413671	8.80	1 (Ref.)			1 (Ref.)			1 (Ref.)		
Increased	22386	2328	220614	10.55	1.138	1.089-1.190	<.001	1.115	1.067-1.166	<.001	1.148	1.098-1.200	<.001

**Myocardial infarction**													
Decreased	13399	235	141153	1.66	0.939	0.821-1.073	0.355	0.956	0.835-1.093	0.508	1.063	0.928-1.219	0.377
Maintained	141351	2559	1450866	1.76	1 (Ref.)			1 (Ref.)			1 (Ref.)		
Increased	22386	441	228160	1.93	1.007	0.910-1.115	0.887	0.972	0.879-1.076	0.589	1.000	0.904-1.107	0.995

CKM, Cardiovascular-Kidney-Metabolic syndrome; CI, confidence interval; HR, hazard ratio; IR, incidence rate; MI, myocardial infarction

Model 1: adjusted by age, sex.

Model 2: adjusted for age, sex, smoking status, alcohol consumption, physical activity, and household income.

Model 3: Adjusted for age, sex, smoking status, alcohol consumption, physical activity, household income, use of antihypertensive drugs, glucose-lowering drugs, lipid-lowering drugs, and antiplatelet drugs.

Table S12. Clinical outcomes according to changes in CKM stage among participants initially at stage 0

					Model 1			Model 2			Model 3		
	Subjects	Events	Person-year	IR	HR	95% CI	p value	HR	95% CI	p value	HR	95% CI	p value
**Composite primary outcome (all-cause death, heart failure, stroke, or myocardial infarction)**													
Maintained	102402	1958	1147016	1.71	1 (Ref.)			1 (Ref.)			1 (Ref.)		
Increased	56007	1949	625417	3.12	1.189	1.116-1.268	<.001	1.247	1.166-1.333	1.333	1.200	1.120-1.286	<.001
**All-cause death**													
Maintained	102402	970	1151054	0.84	1 (Ref.)			1 (Ref.)			1 (Ref.)		
Increased	56007	1008	629605	1.60	1.144	1.046-1.251	0.003	1.252	1.141-1.375	<.001	1.2	1.089-1.322	<.001
**Heart failure**													
Maintained	102402	602	1149241	0.52	1 (Ref.)			1 (Ref.)			1 (Ref.)		
Increased	56007	607	627777	0.97	1.25	1.114-1.402	<.001	1.318	1.169-1.487	<.001	1.241	1.094-1.407	<.001
**Stroke (ischemic or hemorrhagic)**													
Maintained	102402	523	1148913	0.46	1 (Ref.)			1 (Ref.)			1 (Ref.)		
Increased	56007	524	627495	0.84	1.216	1.075-1.376	0.002	1.216	1.069-1.384	0.003	1.175	1.027-1.344	0.019
**Myocardial infarction**													

Maintained	102402	85	1150723	0.07	1 (Ref.)			1 (Ref.)			1 (Ref.)		
Increased	56007	137	628994	0.22	1.772	1.346-2.331	<.001	1.546	1.161-2.058	0.003	1.485	1.106-1.994	0.009

CKM, Cardiovascular-Kidney-Metabolic syndrome; CI, confidence interval; HR, hazard ratio; IR, incidence rate; MI, myocardial infarction

Model 1: adjusted by age, sex.

Model 2: adjusted for age, sex, smoking status, alcohol consumption, physical activity, and household income.

Model 3: Adjusted for age, sex, smoking status, alcohol consumption, physical activity, household income, use of antihypertensive drugs, glucose-lowering drugs, lipid-lowering drugs, and antiplatelet drugs.

Table S13. Clinical outcomes according to changes in CKM stage among participants initially at stage 1

					Model 1			Model 2			Model 3		
	Subjects	Events	Person-year	IR	HR	95% CI	p value	HR	95% CI	p value	HR	95% CI	p value
**Composite primary outcome (all-cause death, heart failure, stroke, or myocardial infarction)**													
Decreased	19816	535	221814	2.41	1.008	0.917-1.108	0.875	0.992	0.895-1.100	0.876	0.995	0.897-1.103	0.921
Maintained	71515	2171	799246	2.72	1 (Ref.)			1 (Ref.)			1 (Ref.)		
Increased	52583	2560	585225	4.37	1.257	1.187-1.332	<.001	1.228	1.159-1.301	<.001	1.175	1.106-1.249	<.001
**All-cause death**													
Decreased	19816	264	222913	1.18	1.098	0.959-1.258	0.176	0.991	0.854-1.149	0.901	0.995	0.858-1.154	0.944
Maintained	71515	993	804095	1.23	1 (Ref.)			1 (Ref.)			1 (Ref.)		
Increased	52583	1141	591676	1.93	1.169	1.073-1.274	<.001	1.142	1.048-1.244	0.003	1.085	0.990-1.188	0.080
**Heart failure**													
Decreased	19816	151	222448	0.68	0.847	0.711-1.010	0.064	0.908	0.751-1.097	0.317	0.912	0.754-1.102	0.340
Maintained	71515	716	801852	0.89	1 (Ref.)			1 (Ref.)			1 (Ref.)		
Increased	52583	839	588893	1.42	1.27	1.148-1.405	<.001	1.243	1.123-1.374	<.001	1.156	1.039-1.287	0.008

**Stroke (ischemic or hemorrhagic)**													
Decreased	19816	144	222346	0.65	0.979	0.816-1.175	0.820	1.002	0.822-1.220	0.987	1.005	0.825-1.224	0.964
Maintained	71515	597	801668	0.74	1 (Ref.)			1 (Ref.)			1 (Ref.)		
Increased	52583	766	588467	1.30	1.36	1.220-1.515	<.001	1.326	1.190-1.478	<.001	1.299	1.160-1.454	<.001
**Myocardial infarction**													
Decreased	19816	37	222770	0.17	1.063	0.741-1.524	0.740	1.318	0.894-1.942	0.164	1.322	0.897-1.949	0.159
Maintained	71515	148	803473	0.18	1 (Ref.)			1 (Ref.)			1 (Ref.)		
Increased	52583	232	590706	0.39	1.64	1.333-2.018	<.001	1.545	1.255-1.902	<.001	1.525	1.230-1.890	<.001

CKM, Cardiovascular-Kidney-Metabolic syndrome; CI, confidence interval; HR, hazard ratio; IR, incidence rate; MI, myocardial infarction

Model 1: adjusted by age, sex.

Model 2: adjusted for age, sex, smoking status, alcohol consumption, physical activity, and household income.

Model 3: Adjusted for age, sex, smoking status, alcohol consumption, physical activity, household income, use of antihypertensive drugs, glucose-lowering drugs, lipid-lowering drugs, and antiplatelet drugs.

Table S14. Clinical outcomes according to changes in CKM stage among participants initially at stage 2

					Model 1			Model 2			Model 3		
	Subjects	Events	Person-year	IR	HR	95% CI	p value	HR	95% CI	p value	HR	95% CI	p value
**Composite primary outcome (all-cause death, heart failure, stroke, or myocardial infarction)**													
Decreased	63231	3043	704598	4.32	0.768	0.740-0.797	<.001	0.768	0.740-0.798	<.001	0.903	0.867-0.940	<.001
Maintained	414351	38314	4494086	8.53	1 (Ref.)			1 (Ref.)			1 (Ref.)		
Increased	24451	6828	241309	28.30	1.594	1.552-1.638	<.001	1.510	1.469-1.552	<.001	1.391	1.353-1.431	<.001
**All-cause death**													
Decreased	63231	1510	711476	2.12	0.955	0.906-1.007	0.088	0.879	0.834-0.928	<.001	0.979	0.924-1.037	0.465
Maintained	414351	16463	4600798	3.58	1 (Ref.)			1 (Ref.)			1 (Ref.)		
Increased	24451	3620	260669	13.89	1.544	1.486-1.604	<.001	1.47	1.414-1.527	<.001	1.35	1.298-1.404	<.001
**Heart failure**													
Decreased	63231	905	708600	1.28	0.633	0.591-0.677	<.001	0.669	0.625-0.716	<.001	0.925	0.860-0.995	0.036
Maintained	414351	14215	4551285	3.12	1 (Ref.)			1 (Ref.)			1 (Ref.)		
Increased	24451	2788	250679	11.12	1.757	1.682-1.834	<.001	1.675	1.603-1.749	<.001	1.51	1.444-1.578	<.001

**Stroke (ischemic or hemorrhagic)**												
Decreased	63231	869	707943	1.23	0.735	0.686-0.788	<.001	0.750	0.699-0.804	<.001	0.861	0.800-0.928	<.001
Maintained	414351	11607	4550442	2.55	1 (Ref.)			1 (Ref.)			1 (Ref.)		
Increased	24451	2083	251560	8.28	1.567	1.491-1.646	<.001	1.481	1.409-1.557	<.001	1.367	1.300-1.438	<.001
**Myocardial infarction**													
Decreased	63231	259	710315	0.36	0.578	0.509-0.656	<.001	0.603	0.531-0.685	<.001	0.676	0.592-0.773	<.001
Maintained	414351	3693	4583538	0.81	1 (Ref.)			1 (Ref.)			1 (Ref.)		
Increased	24451	537	258081	2.08	1.504	1.369-1.654	<.001	1.342	1.220-1.476	<.001	1.24	1.126-1.366	<.001

CKM, Cardiovascular-Kidney-Metabolic syndrome; CI, confidence interval; HR, hazard ratio; IR, incidence rate; MI, myocardial infarction
Model 1: adjusted by age, sex.
Model 2: adjusted for age, sex, smoking status, alcohol consumption, physical activity, and household income.
Model 3: Adjusted for age, sex, smoking status, alcohol consumption, physical activity, household income, use of antihypertensive drugs, glucose-lowering drugs, lipid-lowering drugs, and antiplatelet drugs.

Table S15. Clinical outcomes according to changes in CKM stage among participants initially at stage 3

	Subjects	Events	Person-year	IR	Model 1			Model 2			Model 3		
					HR	95% CI	p value	HR	95% CI	p value	HR	95% CI	p value
**Composite primary outcome (all-cause death, heart failure, stroke, or myocardial infarction)**													
Decreased	13059	3053	130733	23.35	0.779	0.746-0.813	<.001	0.793	0.759-0.828	<.001	0.861	0.823-0.900	<.001
Maintained	33819	17668	286139	61.75	1 (Ref.)			1 (Ref.)			1 (Ref.)		
Increased	1368	970	9185	105.61	1.851	1.735-1.975	<.001	1.863	1.746-1.988	<.001	1.750	1.638-1.869	<.001
**All-cause death**													
Decreased	13059	1794	138097	12.99	0.785	0.744-0.829	<.001	0.790	0.748-0.835	<.001	0.881	0.832-0.932	<.001
Maintained	33819	13182	318826	41.35	1 (Ref.)			1 (Ref.)			1 (Ref.)		
Increased	1368	701	11836	59.22	1.553	1.439-1.676	<.001	1.577	1.461-1.702	<.001	1.496	1.384-1.616	<.001
**Heart failure**													
Decreased	13059	1114	134449	8.29	0.713	0.664-0.766	<.001	0.732	0.682-0.786	<.001	0.798	0.741-0.858	<.001
Maintained	33819	6358	300970	21.13	1 (Ref.)			1 (Ref.)			1 (Ref.)		
Increased	1368	490	10043	48.79	2.561	2.336-2.808	<.001	2.539	2.315-2.784	<.001	2.277	2.073-2.502	<.001

**Stroke (ischemic or hemorrhagic)**												
Decreased	13059	886	134468	6.59	0.759	0.699-0.824	<.001	0.783	0.721-0.850	<.001	0.849	0.780-0.924	<.001
Maintained	33819	4413	303055	14.56	1 (Ref.)			1 (Ref.)			1 (Ref.)		
Increased	1368	302	10769	28.04	1.989	1.770-2.235	<.001	2.01	1.788-2.259	<.001	1.892	1.680-2.131	<.001
**Myocardial infarction**													
Decreased	13059	235	137024	1.72	0.705	0.597-0.832	<.001	0.755	0.639-0.892	0.001	0.821	0.694-0.972	<.001
Maintained	33819	899	315235	2.85	1 (Ref.)			1 (Ref.)			1 (Ref.)		
Increased	1368	54	11639	4.64	1.615	1.227-2.126	<.001	1.61	1.222-2.121	<.001	1.382	1.045-1.828	<.001

CKM, Cardiovascular-Kidney-Metabolic syndrome; CI, confidence interval; HR, hazard ratio; IR, incidence rate; MI, myocardial infarction

Model 1: adjusted by age, sex.

Model 2: adjusted for age, sex, smoking status, alcohol consumption, physical activity, and household income.

Model 3: Adjusted for age, sex, smoking status, alcohol consumption, physical activity, household income, use of antihypertensive drugs, glucose-lowering drugs, lipid-lowering drugs, and antiplatelet drugs.

Figure S1. CKM stage flow between health check-ups by sex and age subgroups.

(A) Men.

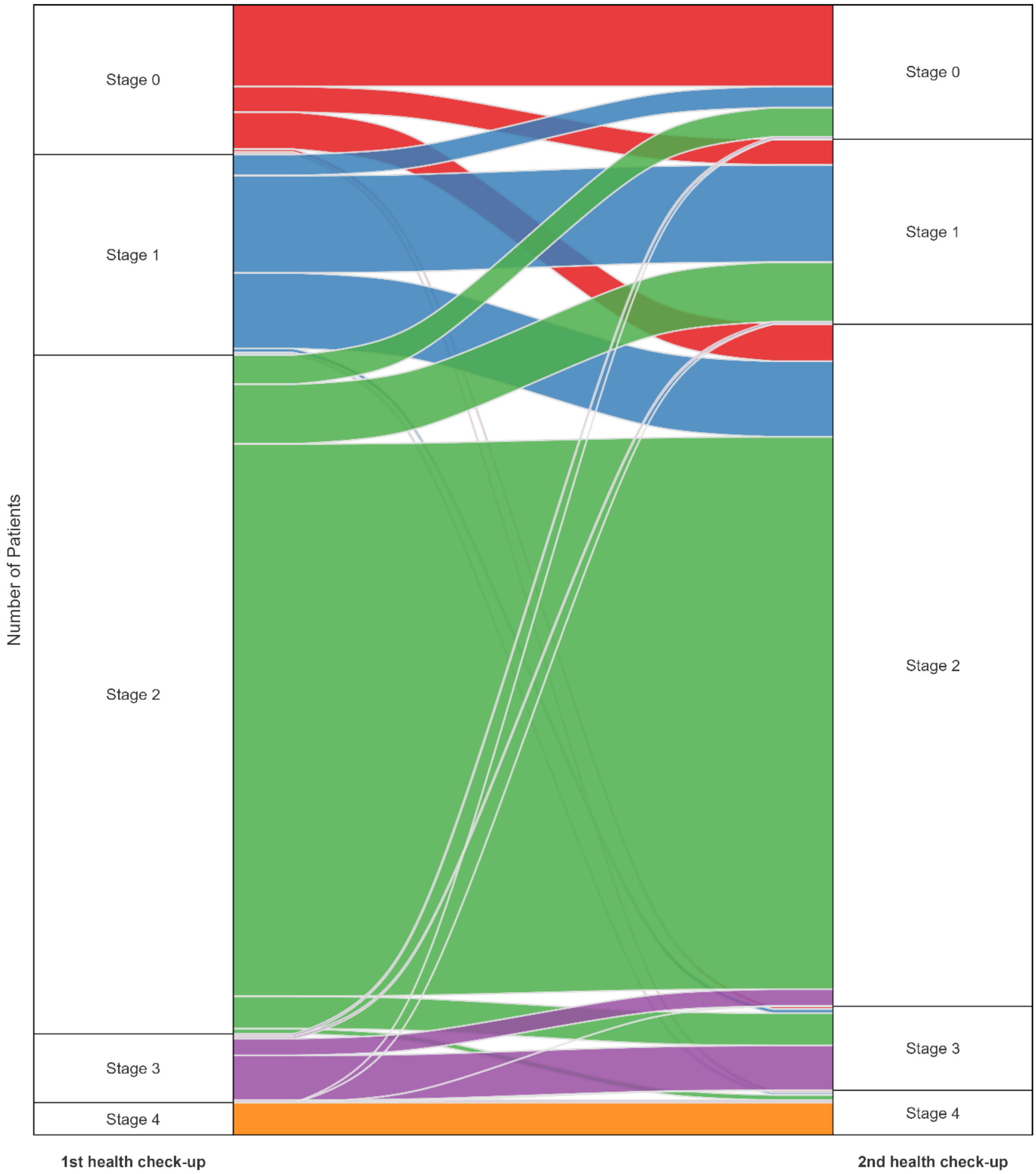

(B) Women.

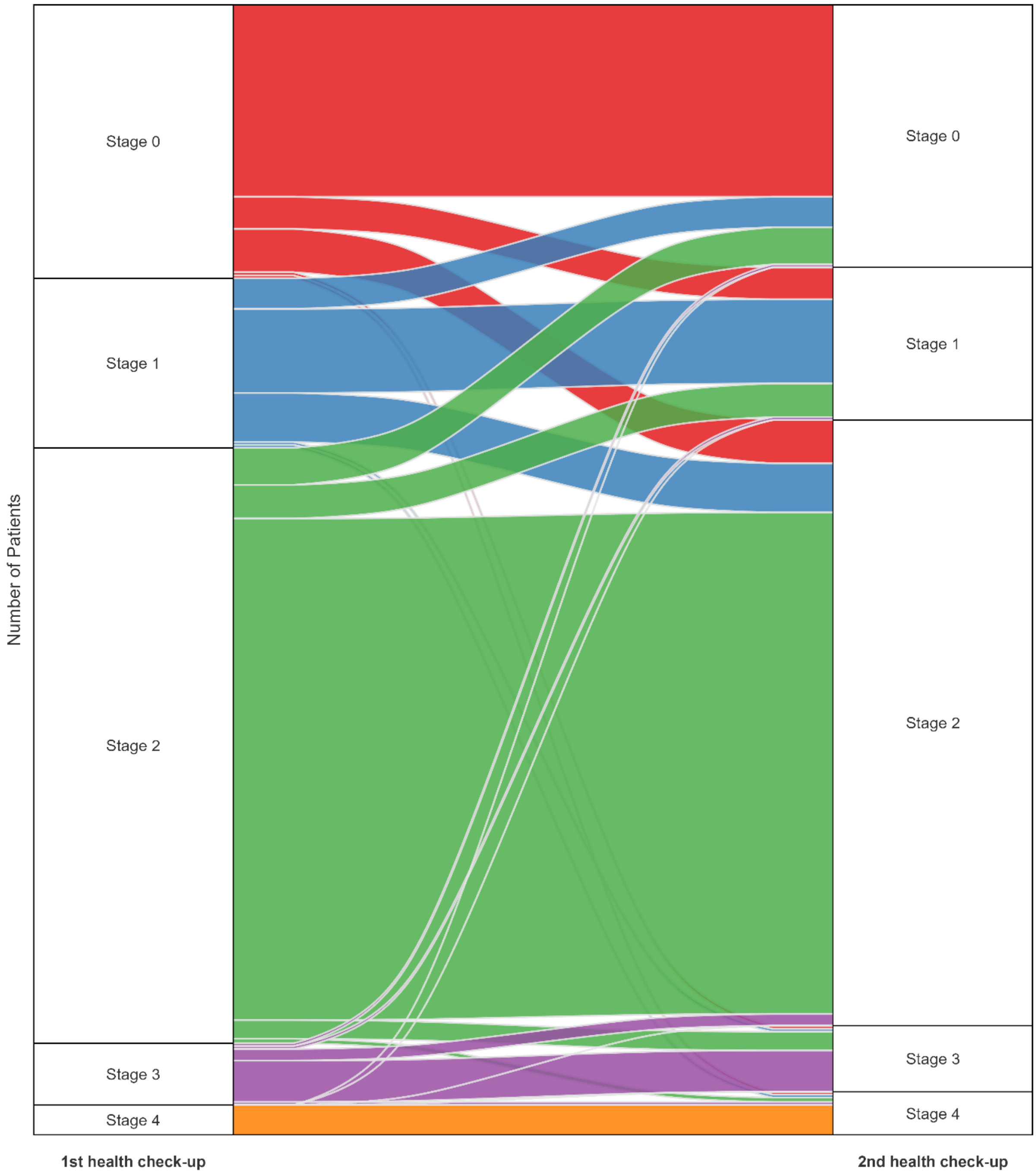

Number of Patients
Stage 0
Stage 1
Stage 2
Stage 3
Stage 4
1st health check-up
Stage 0
Stage 1
Stage 2
Stage 3
Stage 4
2nd health check-up

(C) 20-29 years.

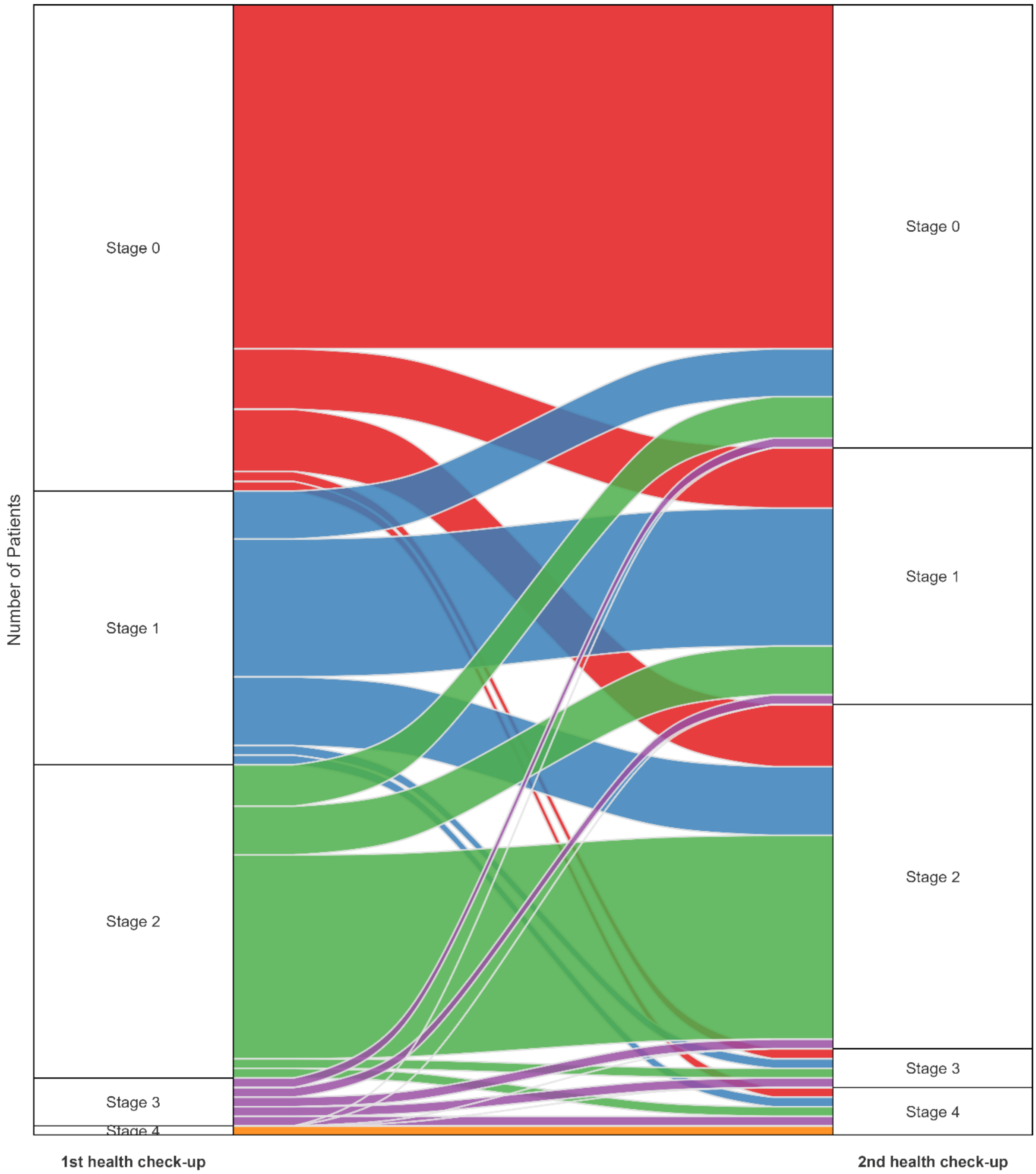

Number of Patients
Stage 0
Stage 1
Stage 2
Stage 3
Stage 4
1st health check-up
Stage 0
Stage 1
Stage 2
Stage 3
Stage 4
2nd health check-up

(D) 30-39 years.

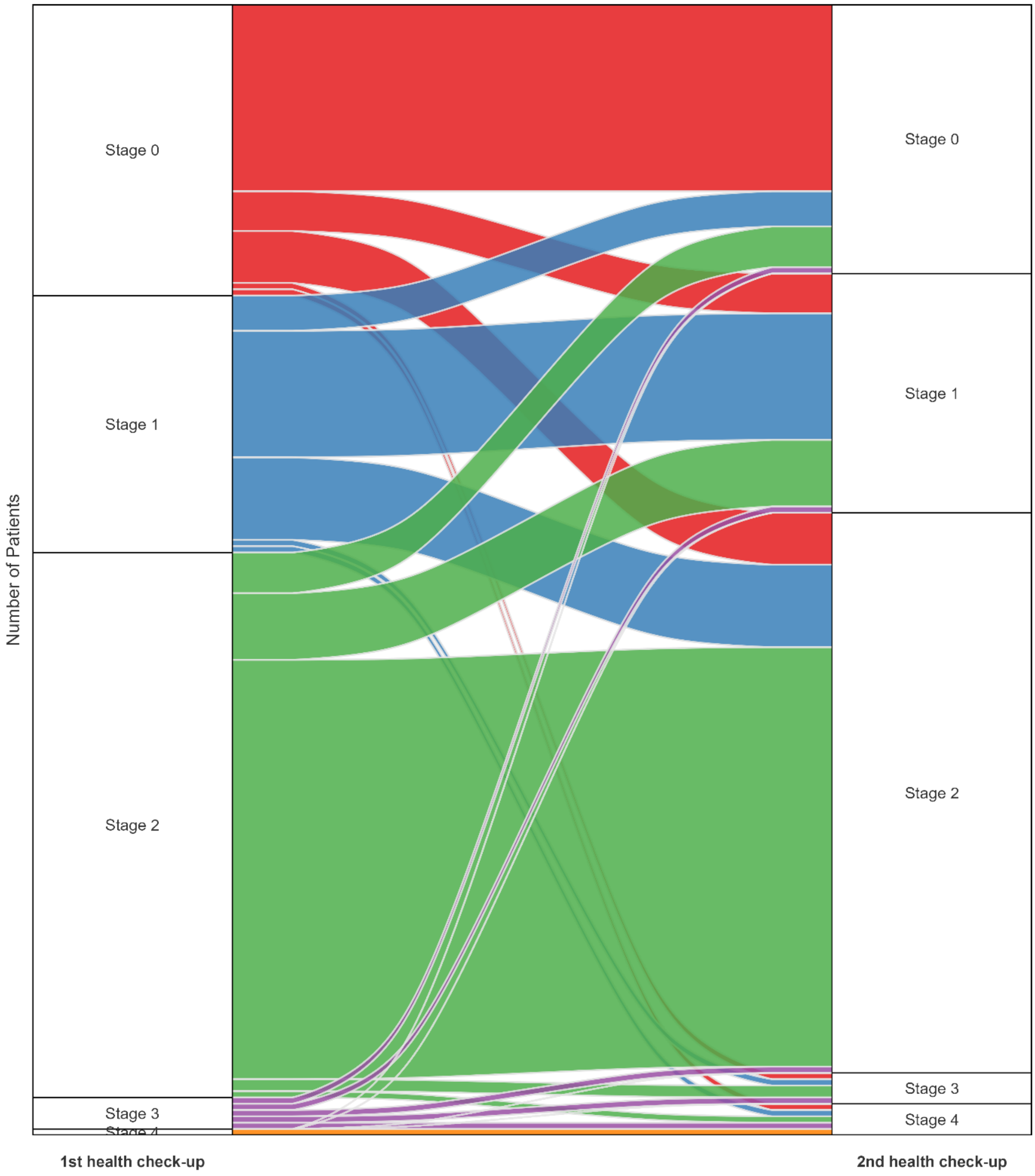

Number of Patients
Stage 0
Stage 1
Stage 2
Stage 3
Stage 4
1st health check-up
Stage 0
Stage 1
Stage 2
Stage 3
Stage 4
2nd health check-up

(E) 40-49 years.

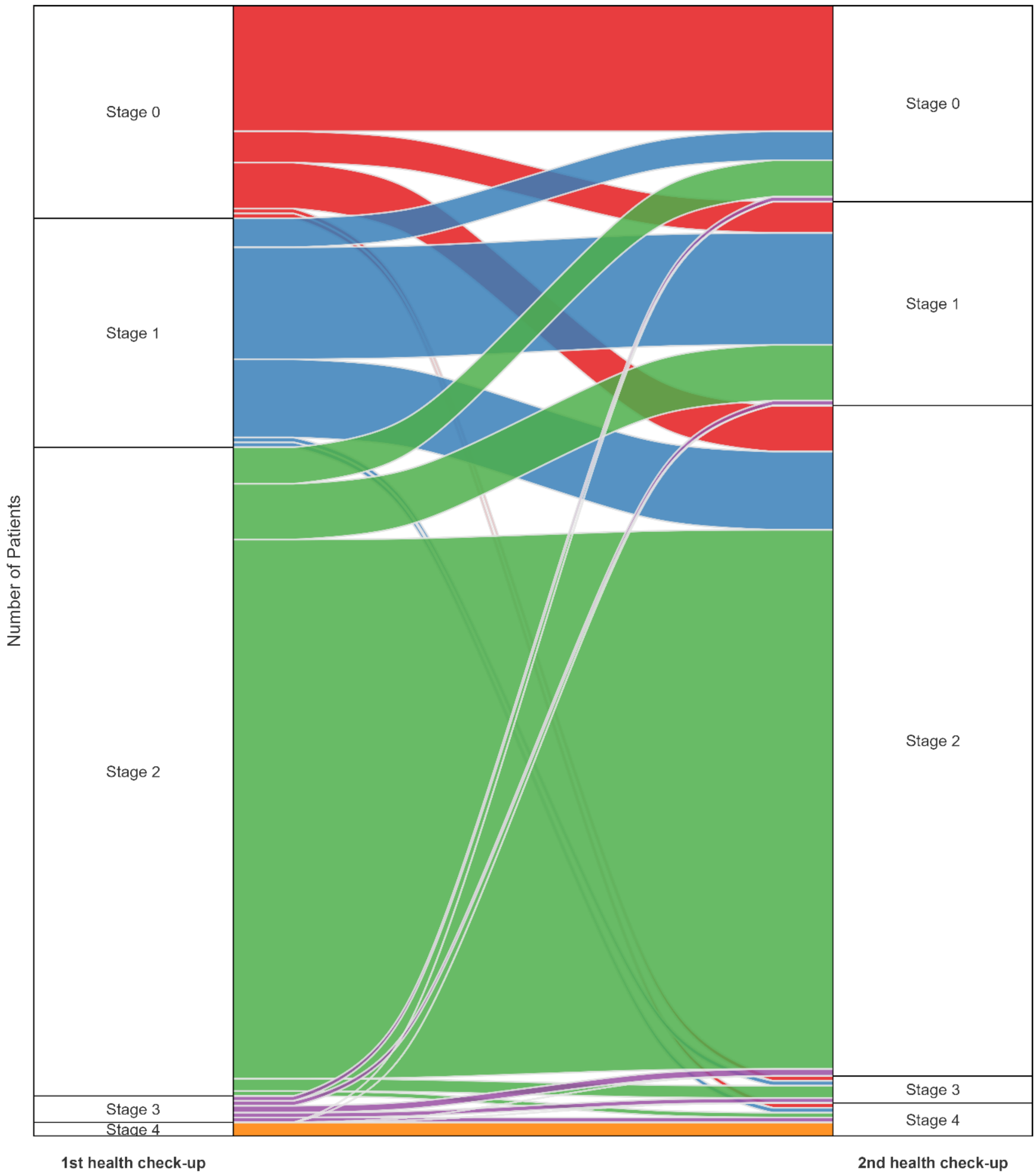
Number of Patients
Stage 0
Stage 1
Stage 2
Stage 3
Stage 4
Stage 0
Stage 1
Stage 2
Stage 3
Stage 4
1st health check-up
2nd health check-up

(F) 50-59 years.

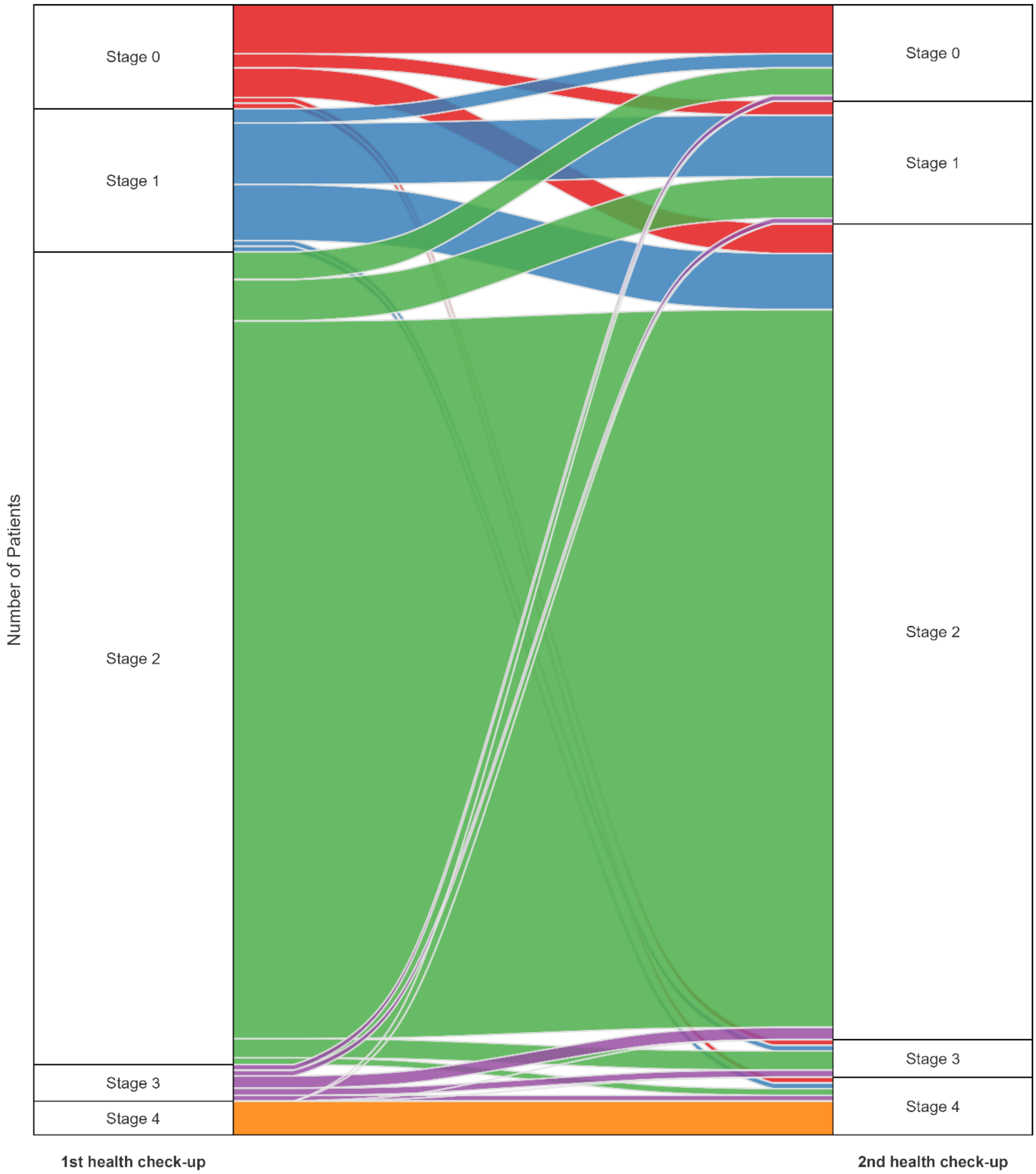
Number of Patients
Stage 0
Stage 1
Stage 2
Stage 3
Stage 4
1st health check-up
Stage 0
Stage 1
Stage 2
Stage 3
Stage 4
2nd health check-up

(G) ≥60 years.

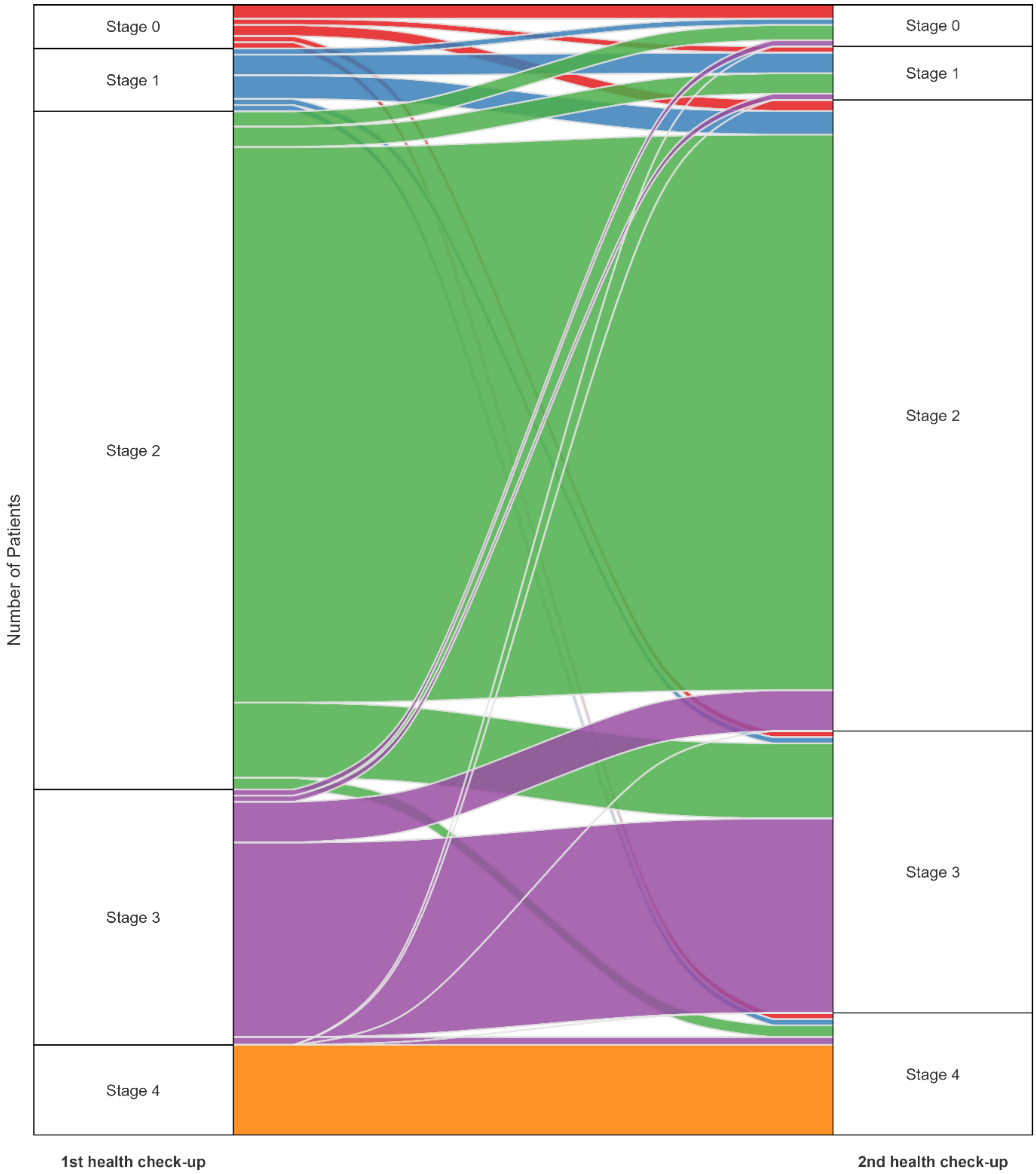

Stage 0
Stage 1
Stage 2
Stage 3
Stage 4
Stage 0
Stage 1
Stage 2
Stage 3
Stage 4
Number of Patients
1st health check-up
2nd health check-up

Figure S2. Kaplan-Meier curve for clinical outcomes according to changes in CKM stage.

(A) Composite primary outcome (all-cause death, heart failure, stroke, or myocardial infarction).

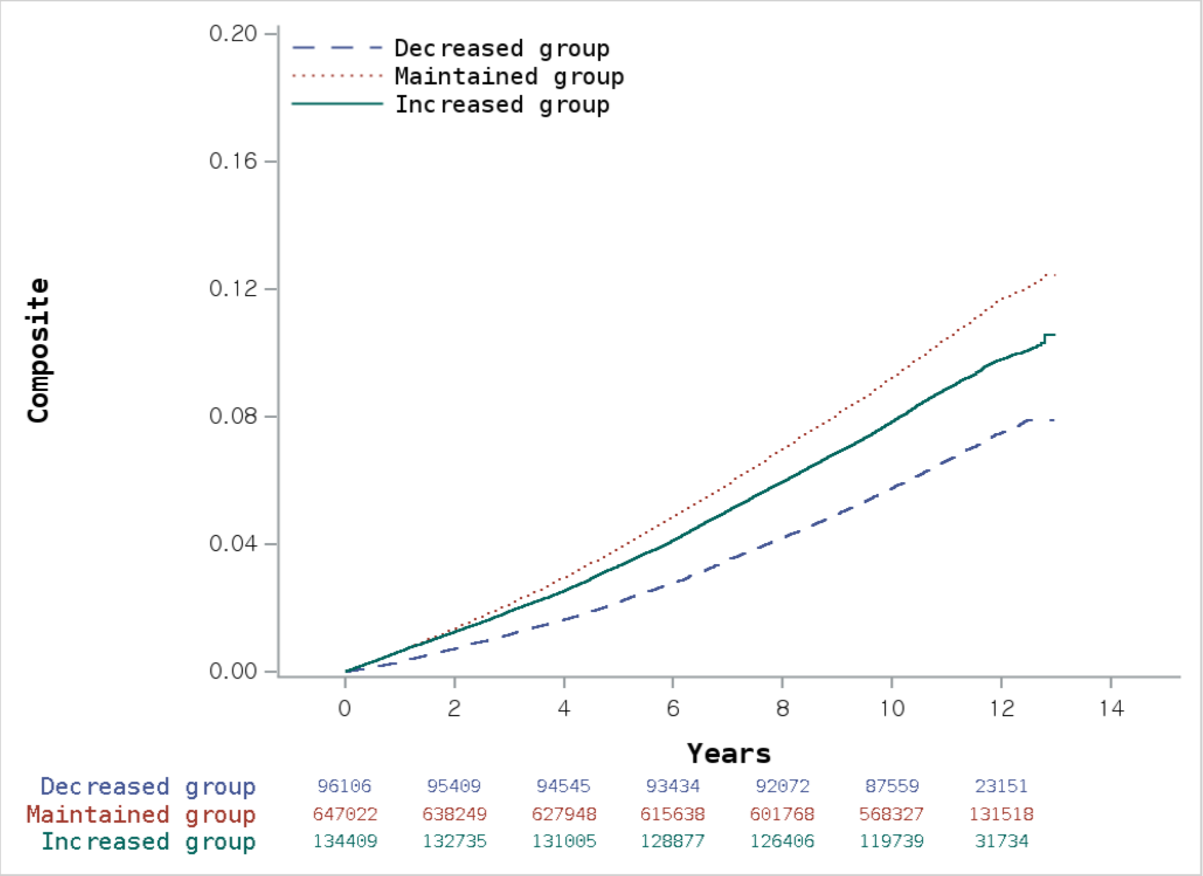

(B) All-cause death.

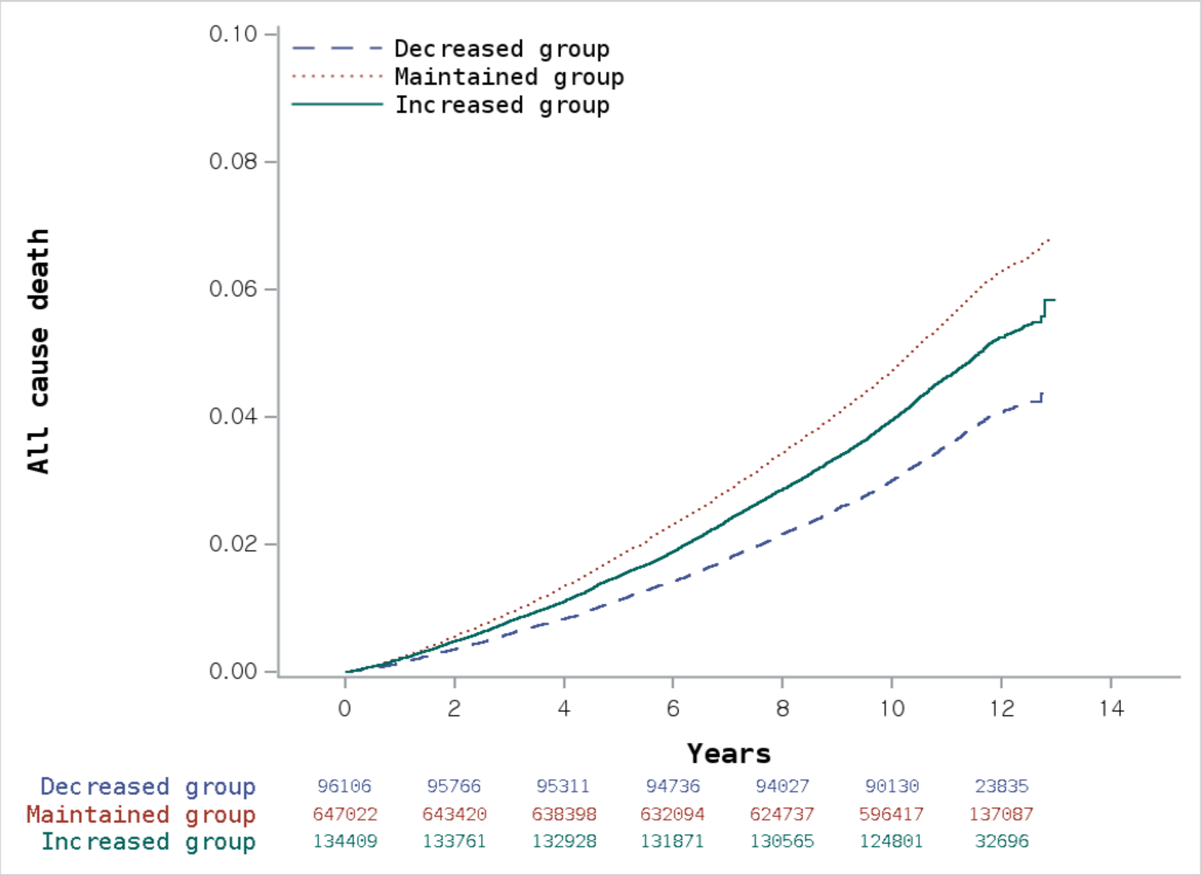

(C) Heart failure.

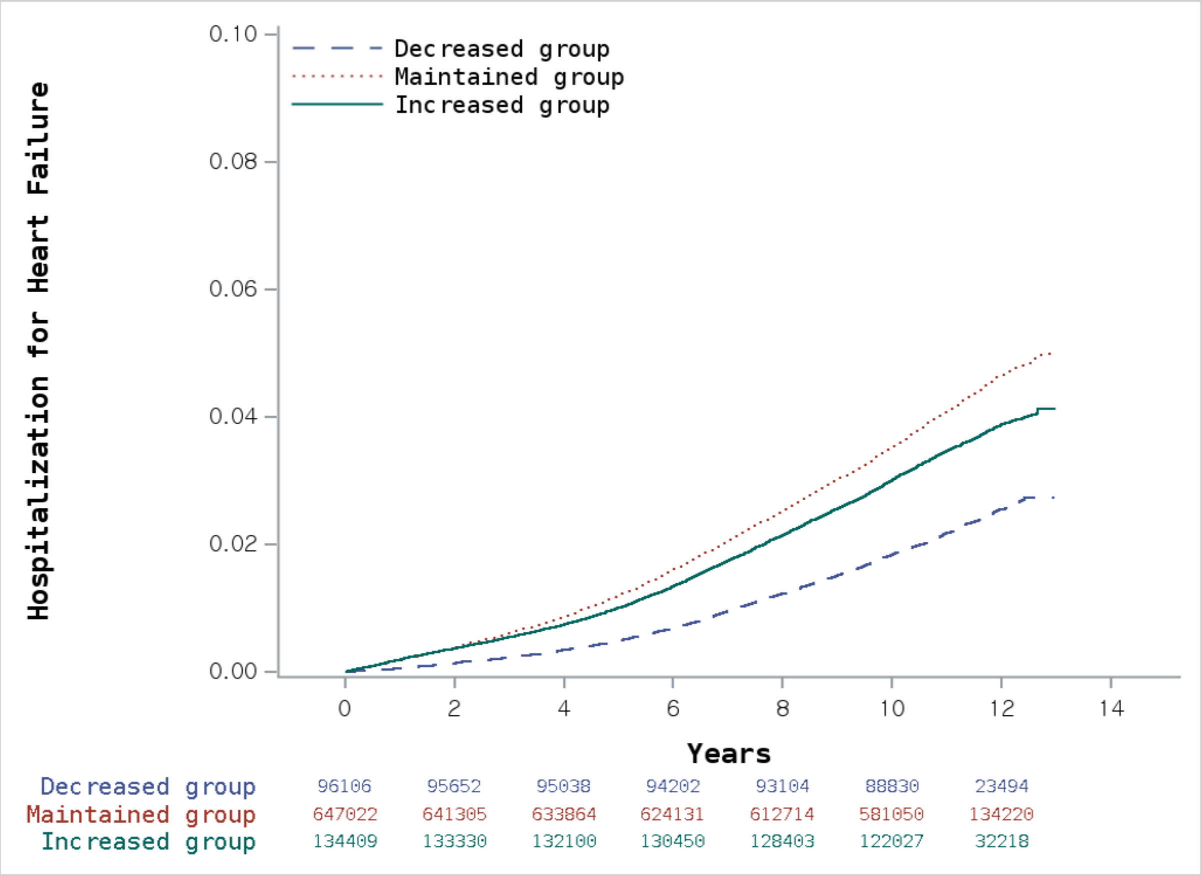

(D) Stroke (ischemic or hemorrhagic).

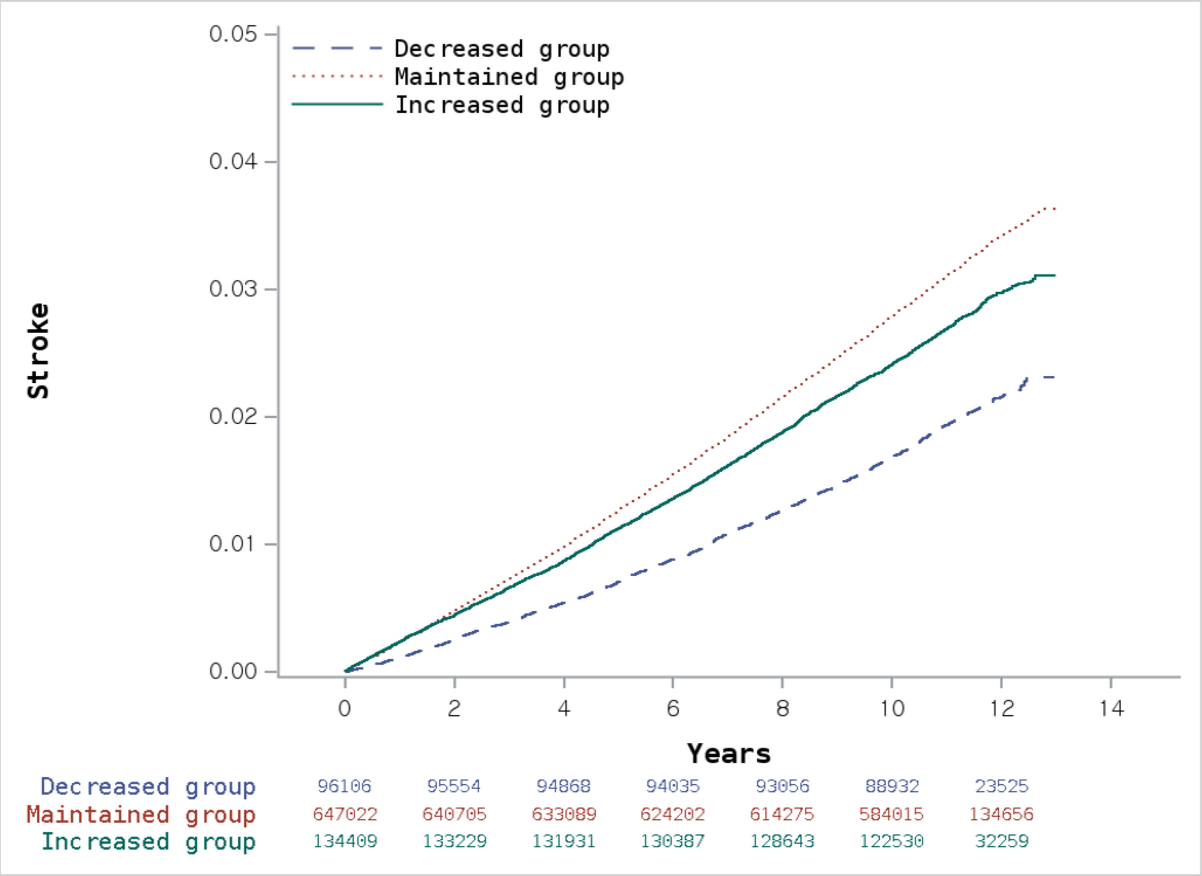

(E) Myocardial infarction.

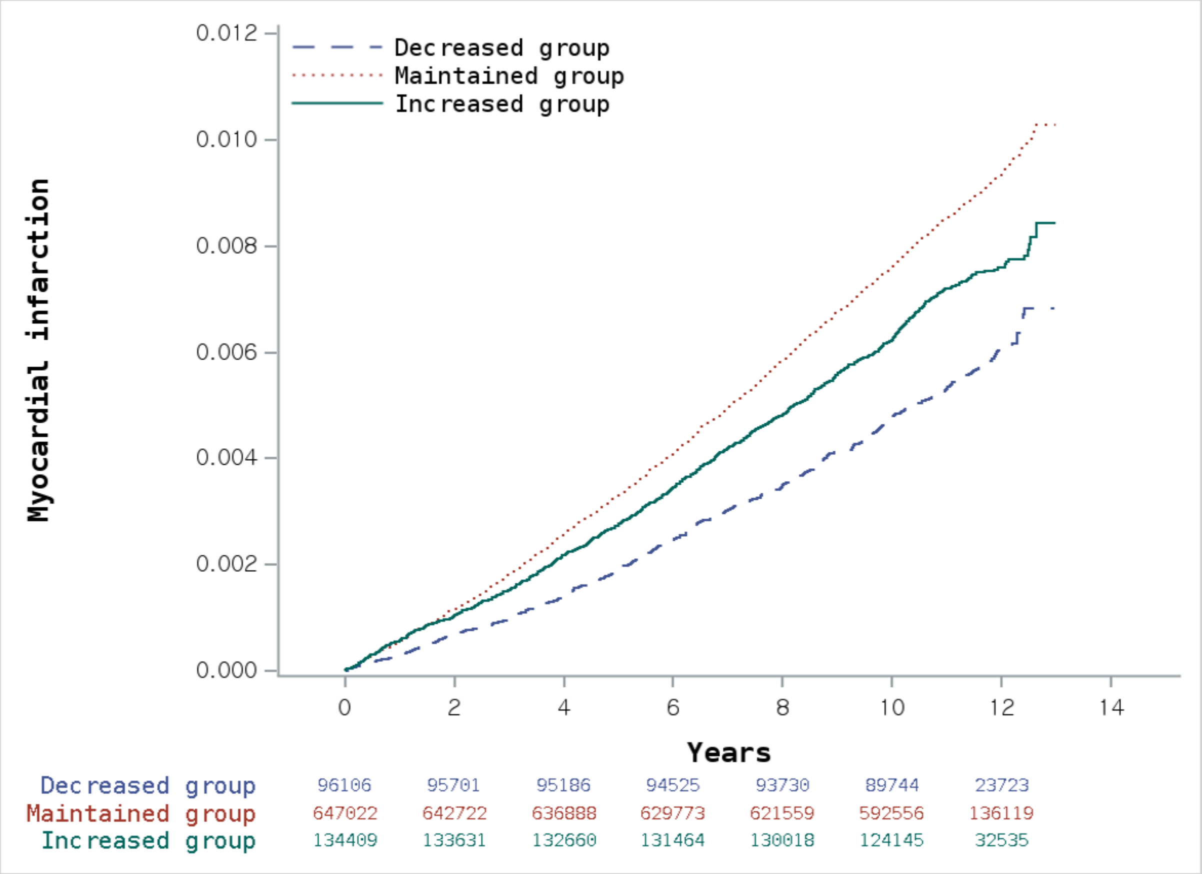
Decreased group
Maintained group
Increased group
Myocardial infarction
0.012
0.010
0.008
0.006
0.004
0.002
0.000
0
2
4
6
8
10
12
14
Years
Decreased group 96106 95701 95186 94525 93730 89744 23723
Maintained group 647022 642722 636888 629773 621559 592556 136119
Increased group 134409 133631 132660 131464 130018 124145 32535
